# Generalized Species Richness Indices for Diversity

**DOI:** 10.3390/e24101504

**Published:** 2022-10-21

**Authors:** Zhiyi Zhang

**Affiliations:** Department of Mathematics and Statistics, University of North Carolina at Charlotte, Charlotte, NC 28223, USA; zzhang@uncc.edu

**Keywords:** diversity index, species richness, generalized species richness, breakdown point, Turing’s formula, sample coverage, 62F10, 62F12, 62G05, 62G20

## Abstract

A generalized notion of species richness is introduced. The generalization embeds the popular index of species richness on the boundary of a family of diversity indices each of which is the number of species in the community after a small proportion of individuals belonging to the least minorities is trimmed. It is established that the generalized species richness indices satisfy a weak version of the usual axioms for diversity indices, are qualitatively robust against small perturbations in the underlying distribution, and are collectively complete with respect to all information of diversity. In addition to a natural plug-in estimator of the generalized species richness, a bias-adjusted estimator is proposed, and its statistical reliability is gauged via bootstrapping. Finally an ecological example and supportive simulation results are given.

## 1. Introduction

Consider an ecological community with a well-defined set of species $X=\{\ell_{k};k=1,\cdots ,K\}$ and an associated distribution of proportions, also known as species abundances, $p=\{p_{k};k=1,\cdots ,K\}$. More generally, X and p may be considered as a countable alphabet and an associated probability distribution, where *K* may be a finite integer or infinite. In this article, the $\ell_{k}$s may be interchangeably referred to as letters of an alphabet or species in a community, and p may be referred to as a species abundance distribution or a probability distribution. The notion of diversity in a community has been of long standing research interest. What is diversity and how should it be quantified have been the two fundamental questions at the center of diversity literature for many decades. A large number of diversity indices have been proposed in the history, for example, those by Simpson in [1], Shannon in [2], Rényi in [3] and Tsallis in [4] are among many most commonly used indices, each of which has been argued to have particular merit. The opinions on diversity and possible numerical indices to measure it are indeed diverse. There are even doubts in the general concept of diversity, for example see [5,6]; and there is also a school of thought which believes that the species richness is the only acceptable diversity index, for example see [7]. There have also been unifying efforts to define diversity indices to accommodate a range of such indices, for example see [8,9,10,11], among others. Nevertheless when it come to measuring diversity, there is a lack of agreement for a generally satisfactory univariate index. The general consensus in the existing literature seems to be that a better description of diversity should be a multidimensional index set, or a profile. A good introduction to diversity profiles is offered in [10] where many basic concepts are articulated and many related references are found.

The departure point of this article is the species richness index, *K*, the number of different species in a community. The species richness index is a part of almost every discussion in the existing literature, and it is so for a good reason. Like the notion of happiness, diversity is an intuitively clear notion for most, but is difficult to quantify. Does there exist a universally accepted index (or an index profile) that would please all? The answer is unknown. If there does, it has not been found. If not, then the objective would be to find one that would have wider acceptance. Either way, the search should and does continue. In that regard, the species richness index *K* is perhaps one of the simplest, the most direct and most intuitive of all existing diversity indices. It is difficult to dismiss such an index.

Nevertheless the species richness index has many weaknesses which can be summarized into the following list.

1. It is oblivious to the magnitude of species abundances.
2. It is ultra-sensitive to redistribution of any arbitrarily small proportion.
3. It is difficult to estimate based on a sample.
4. It does not provide an ordering, or a partial ordering, for communities with infinite number of species.

The first weakness is easily illustrated by a simple example. Consider two distributions with K=2, p={0.5,0.5} and q={0.99,0.01}. The species richness is 2 for both but it clearly does not capture the intuitive notion of diversity. In the diversity literature species richness is sometimes considered a separated type of index from those taking abundances into consideration. This article argues that the separation is not necessary and a slight change of perspective would embed species richness in a profile that naturally takes abundances into consideration.

The second weakness is also easily illustrated by a simple example. Consider p={1−ε,ε} where ε>0 is an arbitrarily small value. The species richness of p is K=2. However taking the abundance $p_{2}=\varepsilon$ and redistributing it to *m* new species, k=2,⋯,m+1, evenly, a new distribution $q=\{q_{1}=1-\varepsilon ,q_{k}=\varepsilon /m;k=2,\cdots ,m+1\}$ is created. It is easily seen first that the species richness of q is K=m+1, second that *m* is arbitrarily large so the species richness of q can be carried over all bounds, and third that the arbitrarily large difference in species richness between p and q is due to an arbitrarily small difference between p and q.

The second weakness demonstrated above is not unique to the species richness. Consider Shannon’s entropy, $H=\sum_{k=1}^{K}p_{k}\ln\left(1/p_{k}\right)$. Taking an arbitrarily small quantity ε>0 (from any $p_{k}$), re-distributing it evenly to *m* new species each of which with proportion ε/m, and hence creating distribution q, it would then add approximately

(1) $$\sum_{i=1}^{m}\frac{\varepsilon }{m}\ln\frac{m}{\varepsilon }=\varepsilon \ln m-\varepsilon \ln\varepsilon$$

to *H* in evaluating entropy of q. (1) may be carried over all bounds as *m* increases indefinitely.

In fact, this issue of ultra-sensitivity is well-known beyond the boundary of diversity literature. In modern data science where the sample space is large, non-metrized, non-ordinal, and not completely pre-scribed, statistical inference often relies on information theoretical quantities that are sensitive to the probabilities of rare events. Such information-theoretic quantities are often ultra-sensitive toward small perturbations in the tail of a distribution.

The third weakness is essentially caused by the second weakness. As demonstrated above, two distributions, different only in the way that one is an arbitrarily stretched version of another by an arbitrarily small mass in abundance, can have arbitrarily different values in species richness. In that regard, in a random sample of size *n*, the species with stretched proportions collectively have very small probability to be represented. This makes it nearly impossible to estimate *K* with any reliability non-parametrically. Estimating *K* with a random sample is a long standing difficult problem in statistics. Interested readers may refer to two excellent survey papers, ref. [12,13], respectively. More specifically, a worthy line of approaches based on Turing’s formula may also be of interest, see [14]. See also for example, [15,16,17,18]. Nevertheless it is fair to say that, not surprisingly, there are no known generally satisfactory estimators of *K*.

The fourth weakness is in the generality of the definition. Generally one would prefer to have a notion of diversity not only for communities with finite *K* but also for K=∞. The species richness does not provide an ordering, or a partial ordering, for all communities with K=∞, In fact, it does not provide an ordering or partial ordering communities with a same K<∞.

The generalized species richness proposed in this article resolves, or at least alleviates all these weaknesses. Toward introducing the generalized species richness indices, consider the second weakness mentioned above once more. Recognizing the fact that an infinitesimal perturbation in the abundance distribution could greatly impact species richness, one may ask the following questions.

1. If 100×α%, where α∈(0,1), of the communities belonging to species with the lowest abundances is trimmed, what would be the species richness of the remaining community?
2. What is the least number of species that can be represented by 100×(1−α)% of the community?

**Figure 1:**
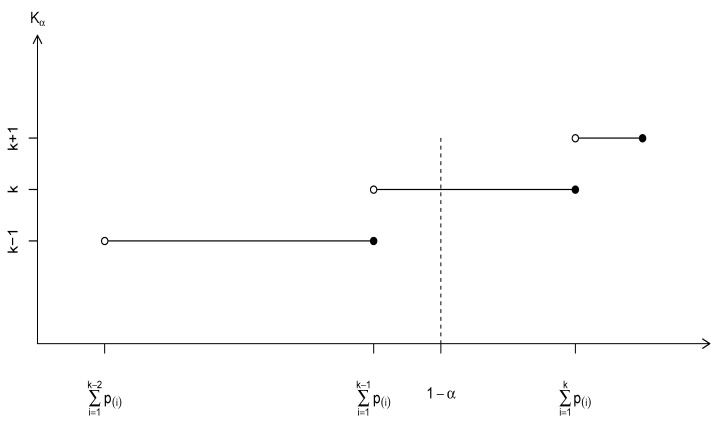
Graphic definition of $K_{\alpha }=k$ given α.

Let the non-increasing ordered $p=\{p_{k};k\geq 1\}$ be denoted by

(2) $$p_{↓}=\left\{p_{\left(k\right)};k\geq 1\right\}$$

where $p_{\left(k\right)}\geq p_{\left(k+1\right)}$ for all k≥1. The answer to both above questions is, for a fixed α∈(0,1),

(3) $$K_{\alpha }=K_{\alpha }\left(p_{↓}\right)=\sum_{k\geq 1}k\times 1\left[\sum_{i=1}^{k-1}p_{\left(i\right)}<1-\alpha \leq \sum_{i=1}^{k}p_{\left(i\right)}\right]$$

(4) $$=\max\left\{k:\sum_{i=1}^{k}p_{\left(i\right)}<1-\alpha \right\}+1$$

(5) $$=\min\left\{k:\sum_{i=1}^{k}p_{\left(i\right)}\geq 1-\alpha \right\}$$

where 1[·] is the indicator function. For a given α∈(0,1), there is only one non-zero term in the summation of (3) with an integer value *k* such that 1−α is sandwiched between $\sum_{i=1}^{k-1}p_{\left(i\right)}$ exclusive and $\sum_{i=1}^{k}p_{\left(i\right)}$ inclusive. See a graphic representation of $K_{\alpha }$ in Figure 1. $K_{\alpha }$ is the proposed generalized species richness, and it may also be reasonably referred to as the α-trimmed species richness. Let

(6) $$K\left(p\right)=\{K_{\alpha }\left(p\right);\alpha \in \left(0,1\right)\}$$

be referred to as the species richness profile.

Revisiting the example of p={0.5,0.5} and q={0.99,0.01} mentioned above for the first weakness of species richness $K=K_{0}$, with say α=0.05, it is easily seen that $K_{0.05}\left(p\right)=2$ and $K_{0.05}\left(q\right)=1$. Revisiting the example of p={1−ε,ε} and its stretched version $q=\{q_{1}=1-\varepsilon ,q_{k}=\varepsilon /m;k=2,\cdots ,m+1\}$ mentioned above for the second weakness of species richness $K=K_{0}$, it is also easy to see that arbitrary stretching of ε, that is, letting *m* increase indefinitely, will not carry $K_{\alpha }\left(q\right)$ over all bounds so long as ε<α. In this regard, it is clear that $K_{\alpha }$ may be viewed as a robustified version of species richness. With the influence from arbitrary stretching of an infinitesimal mass in abundance controlled (but not eliminated), the difficulty level in estimating $K_{\alpha }$ is considerably reduced from that in estimating *K*. Finally the fourth weakness of species richness is eliminated since $K_{\alpha }$ is always finite so long as α>0 for distributions with K<∞ as well as K=∞.

In Section 2, several properties of the generalized species richness are established. More specifically, it is established that every member of K in (6) is a diversity index as it satisfies a weak version of the usual axioms of diversity indices; and a notion of “breakdown point” is introduced and the robustness of $K_{\alpha }$ is gauged accordingly. Furthermore, a notion of “completeness” in profiles is introduced and K of (6), as a profile, is shown to be complete.

To estimate $K_{\alpha }$, let an identically and independently distributed (iid) sample of size *n* be summarized into sample species frequencies, $\left\{Y_{k};k\geq 1\right\}$, and relative species frequencies, $\hat{p}=\left\{{\hat{p}}_{k}=Y_{k}/n;k\geq 1\right\}$; and let ${\hat{p}}_{↓}=\left\{{\hat{p}}_{\left(k\right)};k\geq 1\right\}$ be a non-increasingly ordered $\hat{p}$. A natural estimator of $K_{\alpha }$ is (3), (4) or (5), with ${\hat{p}}_{\left(i\right)}$ in place of $p_{\left(i\right)}$, that is,

(7) $$\begin{array}{cc} {\hat{K}}_{\alpha } & =K_{\alpha }\left({\hat{p}}_{↓}\right)=\sum_{k\geq 1}k\times 1\left[\sum_{i=1}^{k-1}{\hat{p}}_{\left(i\right)}<1-\alpha \leq \sum_{i=1}^{k}{\hat{p}}_{\left(i\right)}\right]\\ \; & =\max\left\{k:\sum_{i=1}^{k}{\hat{p}}_{\left(i\right)}<1-\alpha \right\}+1=\min\left\{k:\sum_{i=1}^{k}{\hat{p}}_{\left(i\right)}\geq 1-\alpha \right\}, \end{array}$$

specifically noting that ${\hat{K}}_{\alpha }$ is based on the same functional $K_{\alpha }\left(\cdot \right)$ in (3) but evaluated at the empirical distribution ${\hat{p}}_{↓}$ instead of $p_{↓}$. It is easy to see that (7) is simply counting the number of species in the sample after 100×α% of the observations in the sample with the lowest (observed) species relative frequencies trimmed. ${\hat{K}}_{\alpha }$ in (7) will be referred to as the plug-in estimator of $K_{\alpha }$ in subsequent text.

However ${\hat{K}}_{\alpha }$ significantly under-estimates $K_{\alpha }$ due to a well-known phenomenon—a perpetual under representation of small probability letters in a finite sample. This phenomenon was perhaps first explicitly identified by Alan Turing during World War II in an effort to break the German naval enigmas, and is referred to as the Turing phenomenon in the subsequent text. The core of the Turing phenomenon is the total probability associated with letters of the alphabet that are not represented in a sample, that is, $\pi_{0}=\sum_{k\geq 1}p_{k}1\left[Y_{k}=0\right]$, also sometimes known as the “missing probability”. In non-parametric estimation of information-theoretic quantities, small probability letters often carry much information and the fact many (possibly infinitely many) of them are missing in a sample often causes a significant downward bias. For example, in view of $\sum_{k\geq 1}w_{k}p_{k}=1$ where $w_{k}=1$ and $p_{k}>0$, Shannon’s entropy $H=\sum_{k\geq 1}\left(\ln\left(1/p_{k}\right)\right)p_{k}$ is an weighted average of $\left\{p_{k}\right\}$ with $w_{k}=\ln\left(1/p_{k}\right)$. For another example, the species richness $K=\sum_{k\geq 1}\left(1/p_{k}\right)p_{k}$ is a weighted average of $\left\{p_{k}\right\}$ with $w_{k}=1/p_{k}$. In both cases, the small probability events get heavy weights and therefore under-representation of them in a sample translates to under-estimation. In comparison of the two examples mentioned above, the Turing phenomenon has a much more profound impact on estimation of *K* than *H* in the sense that (ln(1/p))p→0 and (1/p)p→1 as p→0. Having realized the difficulty in estimating such quantities, it would seem reasonable to device mechanisms, either by modifying the estimands (provided that the modified estimands remain relevant) or the assumption on the underlying distribution, to control the behavior of corresponding estimators. For example, ref. [19,20] discuss certain optimal rates of convergence for a class of estimators of entropy and community size under certain condition to prevent $p_{k}$ from being arbitrarily small, in turn to control the behavior of the estimators. This article however seeks such controls by means of α-trimming, both in the estimand, $K_{\alpha }$, as well as in its estimator, ${\hat{K}}_{\alpha }$, specifically with regard to the notion of species richness.

On the other hand, ${\hat{K}}_{\alpha }$ in (7) may be improved by means of bias correction. There are many possible ways to correct the bias. For simplicity, an estimator based on the basic bootstrap method is proposed as in (14) of Section 3. In the same section, the statistical properties of both $K_{\alpha }\left({\hat{p}}_{↓}\right)$ of (7) and $K_{\alpha }\left({\hat{p}}_{↓}^{♯}\right)$ of (14) are discussed. More specifically several asymptotic properties of partial sums of ${\hat{p}}_{↓}$ are given. Based on these asymptotic results, several conservative one-sample and two-samples inferential procedures regarding the underlying generalized species richness are proposed and justified. Several simulation results are also reported in gauging the performance of the estimators. Finally an real life ecological data set is used to illustrate the proposed method.

The article ends with an appendix where many lemmas, corollaries and propositions, along with their proofs, are found.

## 2. Properties of Generalized Species Richness Indices

Diversity as an intuitive notion is quite clear in most minds. However the quantification of diversity is still quite a distance away from a point of universal consensus. In the diversity literature it is commonly accepted that an index may be reasonably referred to as a diversity index if it satisfies several axioms. For notation convenience, let $P_{K}$ be the family of all distributions such that $K=\sum_{k\geq 1}1\left[p_{k}>0\right]$, that is, on a community with *K* species (or a finite alphabet with cardinality *K*), and let P be the family of all possible distributions on a general countable community. It follows that $P=\cup_{K=1}^{\infty }P_{K}$. Let D(p) be a functional defined for every p∈P. The essential axioms of diversity indices include:

*A*_1_: A diversity index D(p) is invariant under any permutation of species labels, that is, any permutation on the index set {k;k≥1}.
*A*_2_: A diversity index D(p) is minimized at $p=\{p_{\left(1\right)}=1,p_{\left(k\right)}=0;k\geq 2\}$.
*A*_3_: A diversity index D(p) is maximized at $p=\{p_{\left(k\right)}=1/K;k=1,\cdots ,K\}$, the uniform distribution in $P_{K}$ for every positive integer *K*.
*A*_4_: For any distribution p, let $p^{*}$ be the associated distribution of p resulted from a transfer of a mass δ>0 from a higher $p_{i}$ to a lower $p_{j}$ subject to $\delta \leq p_{i}-p_{j}$, with all other $p_{k}$s remain unchanged. A diversity index D(p) satisfies $D\left(p\right)\leq D\left(p^{*}\right)$.

The list of axioms may grow longer representing a more stringent imposition on the underlying diversity indices. There are also stronger versions of the axioms. For example, $A_{2}$ as stated is a weaker version of one that requires the index D(p) to be minimized only at $p=\{p_{\left(1\right)}=1,p_{\left(k\right)}=0;k\geq 2\}$ but not at any other distributions. Similarly $A_{3}$ as stated above also has a stronger version which requires the index D(p) to be maximized only at $p=\{p_{\left(k\right)}=1/K;k=1,\cdots ,K\}$ but not at any other distributions. Axiom $A_{4}$ also has a stronger version which requires a strict inequality, that is, $D\left(p\right)<D\left(p^{*}\right)$. The weaker axioms are chosen in this article because species richness *K*, the reference index of the discussion, satisfies them.

Regardless the length or the version of the axioms, Axiom $A_{1}$ is the most essential of them all and is universally accepted. It is important to recognize the implication of $A_{1}$—every diversity index is a functional of p only through $p_{↓}$. Consequently the domain of all diversity indices can be represented by the subset of P that contains only distributions in non-increasing order, denoted as $P_{↓}$.

For a given α∈(0,1), it is clear $K_{\alpha }$ satisfies $A_{1}$, $A_{2}$ and $A_{3}$. The fact that $K_{\alpha }$ satisfies $A_{4}$ is true but is not so obviously. This fact is one of the main results of this article and is summarized in Proposition A1 along with a lengthy proof, both of which are given in Appendix A. The fact that $K_{\alpha }$ satisfies all axioms $A_{1}$ through $A_{4}$ suggests that it may be reasonably regarded as a diversity index.

To quantify the robustness of the generalized species richness indices against disturbances due to re-distributions of a small abundance (or probability) mass, a notion of breakdown point may be introduced. Breakdown point, roughly speaking, is the greatest proportion of data, whose worst behavior may not carry a function of the data over all bounds. To be more precise, let p∈P be an abundance distribution, let ε∈(0,1) be an arbitrarily small value, and let $\varepsilon_{1}=\left\{\varepsilon_{1,k};k\geq 1\right\}$ and $\varepsilon_{2}=\left\{\varepsilon_{2,k};k\geq 1\right\}$ be two non-negative sequences, each of which is with total mass of ε>0, that is, $\sum_{k\geq 1}\varepsilon_{1,k}=\sum_{k\geq 1}\varepsilon_{2,k}=\varepsilon$. Let

(8) $$p_{\varepsilon }=p-\varepsilon_{1}+\varepsilon_{2}$$

represent a perturbation by subtracting a mass ε away from p by means of $\varepsilon_{1}$ and adding back the same mass by means of $\varepsilon_{2}$.

**Definition** **1.** *Let D(p) be any non-negative function of p∈P. The breakdown point of D at p is*

(9) $$B_{p}\left(D\right)=\sup\left\{\varepsilon :\underset{\varepsilon_{1},\varepsilon_{2}}{\sup}D\left(p_{\varepsilon }\right)<\infty \right\}.$$

Obviously $0\leq B_{p}\left(D\right)\leq 1$. A higher value of $B_{p}\left(D\right)$ is regarded as an indication that *D* is more robust at p.

**Definition** **2.** *Let $B_{p}\left(D\right)$ be as in Definition 1. Let $P_{0}$ be a sub-family of P. For any given α∈(0,1], if $B_{p}\left(D\right)\geq \alpha$ for every $p\in P_{0}$, then D(p) is said to be 100×α% robust with respect to $P_{0}$. In particular, if $B_{p}\left(D\right)\geq \alpha$ for every p∈P, then D(p) is said to be 100×α% robust.*

**Example** **1.** *The species richness, K, is 0%-robust. This is so because $\sup_{\varepsilon }K\left(\left(1-\varepsilon \right)p+\varepsilon \right)=\infty$ for any p∈P and any small ε>0.*

**Example** **2.** *The generalized species richness, $K_{\alpha }$, is 100×α%-robust. This claim is one of the main results of this article and is summarized in Proposition A2. Both the proposition and its proof are given in Appendix A.*

In passing, it may also be of interest to evaluate the robustness of two other community diversity indices, Shannon’s entropy $H=-\sum_{k\geq 1}p_{k}\ln p_{k}$ and the Gini-Simpson index $D=1-\sum_{k\geq 1}p_{k}^{2}$.

**Example** **3.** *Shannon’s entropy is 0%-robust. To see this, for a given p, let ε>0 be an arbitrarily small value and let a total mass of ε>0 cumulatively trimmed from the right end in $p_{↓}=\left\{p_{\left(1\right)},p_{\left(2\right)},\cdots \right\}$, that is, using the language of Definition 1,*

$$\varepsilon_{1}=\left\{0,\cdots ,0,\varepsilon_{K_{\varepsilon }},p_{\left(K_{\varepsilon }+1\right)},p_{\left(K_{\varepsilon }+2\right)},\cdots \right\}$$

*which has zeros in the first $K_{\varepsilon }-1$ positions and $\varepsilon_{K_{\varepsilon }}=\varepsilon -\sum_{i=K_{\varepsilon }+1}^{\infty }p_{\left(i\right)}$ in the $K_{\varepsilon }$ th position. In such a construction, the remainder of the mass of 1−ε covers $K_{\varepsilon }$ species, and $p_{↓}-\varepsilon_{1}=\left\{p_{\left(1\right)},\cdots ,p_{\left(K_{\varepsilon }-1\right)},\sum_{i=1}^{K_{\varepsilon }}p_{\left(i\right)}-\varepsilon ,0,0,\cdots \right\}$. Redistributing the mass ε>0 uniformly over m indices from $i=K_{\varepsilon }+1$ to $i=K_{\varepsilon }+m$ with mass ε/m, resulting in $p_{↓}-\varepsilon_{1}+\varepsilon_{2}=\left\{p_{\left(1\right)},\cdots ,p_{\left(K_{\varepsilon }-1\right)},\sum_{i=1}^{K_{\varepsilon }}p_{\left(i\right)}-\varepsilon ,\varepsilon /m,\cdots ,\varepsilon /m,0,\cdots \right\}$. It follows that, as m→∞,*

$$H\left(p_{↓}-\varepsilon_{1}+\varepsilon_{2}\right)\geq \ln\left(m/\varepsilon \right)\to \infty .$$

**Example** **4.** *The Gini-Simpson index is 100%-robust. This is clearly true because 0<D(p)≤1 for any abundance distribution p∈P.*

A diversity profile is a set of diversity indices containing more than one index. A profile is generally preferred over a single diversity index because it is commonly accepted that diversity is a multi-dimensional notion and is better captured by a multivariate index. An immediate question naturally arises: how much diversity information is contained in a profile? This question can be partially answered with a notion of completeness defined below.

**Definition** **3.** *A profile of indices, $D_{p}=\left\{D_{\alpha }\left(p\right);\alpha \in A\right\}$ where A is a set containing more than one element, is said to be complete, if, for any two distributions p and q, $p_{↓}=q_{↓}$ if and only if $D_{\alpha }\left(p\right)=D_{\alpha }\left(q\right)$ for every α∈A.*

Definition 3 essentially says that a complete profile $D_{p}$ uniquely determines $p_{↓}$, and in turn uniquely determines any other diversity index evaluated at $p_{↓}$.

**Example** **5.** *K(p) of (6) is complete. This claim is clearly true noting, for each positive integer i, $p_{\left(i\right)}=\max\left\{\alpha :K_{\alpha }\left(p_{↓}\right)=i\right\}-\max\left\{\alpha :K_{\alpha }\left(p_{↓}\right)=i-1\right\}$.*

K(p) of (6) is not the only complete profile. The two well known families of diversity indices given in the following two examples are also complete.

**Example** **6.** *The generalized Simpson’s diversity indices, $D\left(p\right)=\{D_{u}\left(p\right)=1-\sum_{k\geq 1}p_{k}^{u};u\geq 1\}$, is complete. The fact that D(p), indexed by positive integers u≥1, is a family of diversity indices is established by Grabchak, Marcon, Lang and Zhang (2017). The claim of completeness follows the fact that $\eta =\{\sum_{k\geq 1}p_{k}^{u};u\geq 1\}$ uniquely determines $p_{↓}$, a fact established in [21].*

**Example** **7.** Rényi’s diversity profile $H\left(p\right)=\{H_{\alpha }\left(p\right)={\left(1-\alpha \right)}^{-1}\ln\left(\sum_{k\geq 1}p_{k}^{\alpha }\right);\alpha \in \left(0,1\right)\cup \left(1,\infty \right)\}$ is complete. The completeness follows the fact that the subset of H(p), $H^{*}\left(p\right)=\left\{H_{u}\left(p\right)={\left(1-u\right)}^{-1}\ln\left(\sum_{k\geq 1}p_{k}^{u}\right);u\geq 1\right\}$, uniquely determines $\eta =\{\sum_{k\geq 1}p_{k}^{u};u\geq 1\}$, which uniquely determines $p_{↓}$.

## 3. Inference

Let the discussion of this section begin with a natural estimator of $K_{\alpha }$, ${\hat{K}}_{\alpha }=K_{\alpha }\left({\hat{p}}_{↓}\right)$, as given in (7), which may be viewed an estimator based on the right-tail of ${\hat{p}}_{↓}$ being trimmed by a fixed mass α. This estimator however presents several difficulties in developing valid inferential procedures regarding $K_{\alpha }$. Towards describing some of these difficulties, the following proposition is first stated and proved.

**Proposition** **1.** *Let $p=\{p_{k};k\geq 1\}$ be the underlying distribution on a countable alphabet, satisfying $p_{k}\geq p_{k+1}$ for every k≥1, let $\hat{p}=\left\{{\hat{p}}_{i};i\geq 1\right\}$ be the corresponding relative letter frequencies in an iid sample of size n, and let $K^{\prime }$ be a positive integer such that $1\leq K^{\prime }<K$. Suppose the multiplicity of $p_{K^{\prime }}$ in p is one. Then as n→∞,*

1. *$\sqrt{n}\left(\sum_{i=1}^{K^{\prime }}{\hat{p}}_{i}-\sum_{i=1}^{K^{\prime }}p_{i}\right)\overset{D}{⟶}N\left(0,\sum_{i=1}^{K^{\prime }}p_{i}\left(1-\sum_{i=1}^{K^{\prime }}p_{i}\right)\right)$;*
2. *$P\left(\sqrt{n}\left(\sum_{i=1}^{K^{\prime }}{\hat{p}}_{\left(i\right)}-\sum_{i=1}^{K^{\prime }}{\hat{p}}_{i}=0\right)\right)\to 0$; and*
3. *$\sqrt{n}\left(\sum_{i=1}^{K^{\prime }}{\hat{p}}_{\left(i\right)}-\sum_{i=1}^{K^{\prime }}p_{i}\right)\overset{D}{⟶}N\left(0,\sum_{i=1}^{K^{\prime }}p_{i}\left(1-\sum_{i=1}^{K^{\prime }}p_{i}\right)\right)$.*

**Proof.**  Part 1 directly follows from the central limit theorem. For Part 2, first consider an aggregation of the letters as follows. If K<∞ let $K^{\prime \prime }=K$, and if K=∞ let $K^{\prime \prime }$ be any index such that $p_{K^{\prime \prime }}^{*}=\sum_{i=K^{\prime \prime }}^{\infty }p_{i}<p_{K^{\prime }}$. Let the observed relative letter frequencies in the sample be aggregated accordingly, in particularly let ${\hat{p}}_{K^{\prime \prime }}^{*}=\sum_{i=K^{\prime \prime }}^{\infty }{\hat{p}}_{i}$. Let ${\hat{p}}^{*}=\left\{{\hat{p}}_{1},\cdots ,{\hat{p}}_{K^{\prime }},\cdots ,{\hat{p}}_{K^{\prime \prime }-1},{\hat{p}}_{K^{\prime \prime }}\right\}$, and let $p^{*}=\left\{p_{1},\cdots ,p_{K^{\prime }},\cdots ,p_{K^{\prime \prime }-1},p_{K^{\prime \prime }}\right\}$. It follows that ${\hat{p}}^{*}\overset{p}{\to }p^{*}$, that is to say that, $P({\hat{p}}^{*}\in n_{\varepsilon }\left(p^{*}\right))\to 1$ where $n_{\varepsilon }\left(p^{*}\right)$ is an arbitrarily small ε-neighborhood centered at the point $p^{*}$. Noting $p_{k}$s are arranged in a non-increasing order, $p_{K^{\prime }}$ has multiplicity 1, $K^{\prime \prime }$ is finite, and $n_{\varepsilon }\left(p^{*}\right)$ is arbitrarily small, the event $\left\{{\hat{p}}^{*}\in n_{\varepsilon }\left(p^{*}\right)\right\}$ implies the event that the set of $K^{\prime }$ largest $\hat{p}$s are identical to the first $K^{\prime }$$\hat{p}$s in $\hat{p}$, that is, $O_{n}\left(K^{\prime }\right)=\left\{\left\{{\hat{p}}_{i};i=1,\cdots ,K^{\prime }\right\}=\left\{{\hat{p}}_{\left(i\right)};i=1,\cdots ,K^{\prime }\right\}\right\}$. It follows that $P(O_{n}\left(K^{\prime }\right))\to 1$, and that for any ε>0.

$$\begin{array}{cc} P & \left(\left|\sqrt{n}\left(\sum_{i=1}^{K^{\prime }}{\hat{p}}_{\left(i\right)}-\sum_{i=1}^{K^{\prime }}{\hat{p}}_{i}\right)\right|>\varepsilon \right)=P\left(\left.\left|\sqrt{n}\left(\sum_{i=1}^{K^{\prime }}{\hat{p}}_{\left(i\right)}-\sum_{i=1}^{K^{\prime }}{\hat{p}}_{i}\right)\right|>\varepsilon \right|O_{n}\left(K^{\prime }\right)\right)P\left(O_{n}\left(K^{\prime }\right)\right)\\ \; & +P\left(\left.\left|\sqrt{n}\left(\sum_{i=1}^{K^{\prime }}{\hat{p}}_{\left(i\right)}-\sum_{i=1}^{K^{\prime }}{\hat{p}}_{i}\right)\right|>\varepsilon \right|O_{n}^{c}\left(K^{\prime }\right)\right)P\left(O_{n}^{c}\left(K^{\prime }\right)\right)\\ \; & =0\times P\left(O_{n}\left(K^{\prime }\right)\right)+P\left(\left.\left|\sqrt{n}\left(\sum_{i=1}^{K^{\prime }}{\hat{p}}_{\left(i\right)}-\sum_{i=1}^{K^{\prime }}{\hat{p}}_{i}\right)\right|>\varepsilon \right|O_{n}^{c}\left(K^{\prime }\right)\right)P\left(O_{n}^{c}\left(K^{\prime }\right)\right)\\ \; & \leq P(O_{n}^{c}\left(K^{\prime }\right))\to 0. \end{array}$$

Part 2 follows.

For Part 3, since

$$\begin{array}{cc} \sqrt{n}\left(\sum_{i=1}^{K^{\prime }}{\hat{p}}_{\left(i\right)}-\sum_{i=1}^{K^{\prime }}p_{i}\right) & =\sqrt{n}\left(\sum_{i=1}^{K^{\prime }}{\hat{p}}_{\left(i\right)}-\sum_{i=1}^{K^{\prime }}{\hat{p}}_{i}\right)+\sqrt{n}\left(\sum_{i=1}^{K^{\prime }}{\hat{p}}_{i}-\sum_{i=1}^{K^{\prime }}p_{i}\right), \end{array}$$

and the first term converges to zero in probability by Part 2, the asymptotic normality follows Part 1 by Slusky’s theorem. □

The first difficulty of ${\hat{K}}_{\alpha }=K_{\alpha }\left({\hat{p}}_{↓}\right)$ is that it cannot be guaranteed to be consistent under general conditions. To see this, one needs only to consider a special case of $\sum_{i\leq K_{\alpha }}p_{k}=1-\alpha$. By Part 3 of Proposition 1, for sufficiently large *n*,

(10) $$\begin{array}{cc} P & \left(\sqrt{n}\left(\sum_{i=1}^{K_{\alpha }}{\hat{p}}_{\left(i\right)}-\sum_{i=1}^{K_{\alpha }}p_{i}\right)>0\right)=P\left(\sum_{i=1}^{K_{\alpha }}{\hat{p}}_{\left(i\right)}-\sum_{i=1}^{K_{\alpha }}p_{i}>0\right)=P\left(\sum_{i=1}^{K_{\alpha }}{\hat{p}}_{\left(i\right)}>1-\alpha \right)\\ \; & =P({\hat{K}}_{\alpha }\geq K_{\alpha }+1)\approx 0.5>0. \end{array}$$

(10) implies inconsistency and, in addition to that, (10) also suggests that, for sufficiently large *n*, ${\hat{K}}_{\alpha }$ could over-estimate $K_{\alpha }$, albeit by at most one. Clearly the said inconsistency is caused by the discrete nature of the functional $K_{\alpha }\left({\hat{p}}_{↓}\right)$.

The second difficulty of ${\hat{K}}_{\alpha }=K_{\alpha }\left({\hat{p}}_{↓}\right)$ is its significant downward bias when *n* is relatively small. To illustrate the bias, consider the extreme case of α=0 in $K_{\alpha }$, which is simply the species richness index, *K*, in case of a finite sample space. If *K* is relatively large, a relatively small iid sample of size *n* would likely not cover all *K* species in the community. In fact, the sample would typically miss a large number of species, that is, $K_{obs}\ll K$ where $K_{obs}$ is observed number of species in a sample. Consequently the empirical distribution, $\hat{p}=\left\{{\hat{p}}_{k};k=1,\cdots ,K\right\}$ would consist of mostly zeros and hence would severely under-represent $p=\{p_{k};k=1,\cdots ,K\}$ in terms of species richness. When α>0 but small, the same qualitative argument explains the significant downward bias of ${\hat{K}}_{\alpha }$.

The possible inconsistency, along with the persistent and significant downward bias, gives much difficulty in developing inferential procedures under general conditions based on asymptotic properties such as Part 3 of Proposition 1.

Next consider bootstrapping 100×(1−β)% confidence intervals (in general standard notions), respectively, of the quantile method $\left[{\hat{\theta }}_{\beta /2}^{*},{\hat{\theta }}_{1-\beta /2}^{*}\right]$ and of the centered quantile method, also known as the basic method, $\left[2\hat{\theta }-{\hat{\theta }}_{1-\beta /2}^{*},2\hat{\theta }-{\hat{\theta }}_{\beta /2}^{*}\right]$, where $\hat{\theta }$ denotes the estimator based on the original sample of size *n* and ${\hat{\theta }}_{1-\beta /2}^{*}$ and ${\hat{\theta }}_{\beta /2}^{*}$, respectively, denote the 100×(1−β/2) th and 100×β/2 th percentiles of the bootstrapping samples.

First let it be noted that the quantile method $\left[{\hat{\theta }}_{\beta /2}^{*},{\hat{\theta }}_{1-\beta /2}^{*}\right]$ is an inadequate 100×(1−β)% confidence. To see this, let the extreme case of $K_{\alpha }=K$ with α=0 be considered once again. There, given an empirical distribution, ${\hat{p}}_{↓}=\left\{{\hat{p}}_{\left(1\right)},{\hat{p}}_{\left(2\right)},\cdots \right\}$. It is clear that ${\hat{K}}_{\alpha }\ll K_{\alpha }$ as already argued above. For the same reason, by sampling from ${\hat{p}}_{↓}$, every ${\hat{K}}_{\alpha }^{*}\leq {\hat{K}}_{\alpha }\ll K_{\alpha }$. Consequently $\left[{\hat{\theta }}_{\beta /2}^{*},{\hat{\theta }}_{1-\beta /2}^{*}\right]$ necessarily excludes $K_{\alpha }$ far to the right, causing the coverage of the bootstrapping interval to have much lower coverage than 1−β. This is to say that, in terms of estimating $K_{\alpha }$, the downward bias of ${\hat{K}}_{\alpha }$ strikes twice in bootstrapping with the quantile method, once in using the original sample and once in using a bootstrapping sample. In fact, it is commonly observed with real data sets that

(11) $${\hat{K}}_{\alpha ,\beta /2}^{*}<{\hat{K}}_{\alpha ,1-\beta /2}^{*}\ll {\hat{K}}_{\alpha }\ll K_{\alpha },$$

where ${\hat{K}}_{\alpha ,\beta /2}^{*}$ and ${\hat{K}}_{\alpha ,1-\beta /2}^{*}$ are the 100×(1−β/2) th and 100×β/2 th percentiles of the estimates of ${\hat{K}}_{\alpha }$ based on bootstrapping samples. See Example 8 below. The discomforting (11) essentially disqualifies the bootstrapping confidence interval based on the quantile method as a valid inferential tool.

However bootstrapping based on the centered quantile method, also known as the basic bootstrapping method, is qualitatively different. There the downward bias ${\hat{K}}_{\alpha }-K_{\alpha }$ is off set by the bootstrapping downward bias ${\hat{K}}_{\alpha }^{*}-{\hat{K}}_{\alpha }$. Once again in the extreme case of $K_{\alpha }=K$ with α=0, since ${\hat{K}}_{\alpha }^{*}\leq {\hat{K}}_{\alpha }$ for every bootstrapping sample, it follows that ${\hat{K}}_{\alpha }-{\hat{K}}_{\alpha ,\beta /2}^{*}\geq {\hat{K}}_{\alpha }-{\hat{K}}_{\alpha ,1-\beta /2}^{*}\geq 0$ and hence ${\hat{K}}_{\alpha }\leq {\hat{K}}_{\alpha }+\left({\hat{K}}_{\alpha }-{\hat{K}}_{\alpha ,1-\beta /2}^{*}\right)\leq {\hat{K}}_{\alpha }+\left({\hat{K}}_{\alpha }-{\hat{K}}_{\alpha ,\beta /2}^{*}\right)$, or

(12) $${\hat{K}}_{\alpha }\leq 2{\hat{K}}_{\alpha }-{\hat{K}}_{\alpha ,1-\beta /2}^{*}\leq 2{\hat{K}}_{\alpha }-{\hat{K}}_{\alpha ,\beta /2}^{*},$$

that is, the centered bootstrapping confidence interval excludes ${\hat{K}}_{\alpha }$ to the left of the interval. In fact (12) is commonly observed with real data sets even when α>0 is small. See Example 8 below. Unlike (11), the fact that ${\hat{K}}_{\alpha }$ is outside of the centered bootstrapping confidence interval in (12) only indicates inadequacy of the estimator ${\hat{K}}_{\alpha }$ but not that of the interval itself. In fact the centered bootstrapping confidence interval,

(13) $$[2{\hat{K}}_{\alpha }-{\hat{K}}_{\alpha ,1-\beta /2}^{*},\;2{\hat{K}}_{\alpha }-{\hat{K}}_{\alpha ,\beta /2}^{*}],$$

represents a bias-adjustment in the right direction, that is, the bias in ${\hat{K}}_{\alpha }$ as an estimator of $K_{\alpha }$ is partially offset by that in ${\hat{K}}_{\alpha }^{*}$ as an estimator of ${\hat{K}}_{\alpha }$. It is to be noted that (12) suggests a bias-adjusted alternative estimator to ${\hat{K}}_{\alpha }$,

(14) $${\hat{K}}_{\alpha }^{♯}=2{\hat{K}}_{\alpha }-{\hat{K}}_{\alpha ,1/2}^{*},$$

where ${\hat{K}}_{\alpha ,1/2}^{*}$ is the median of bootstrapped estimates.

The 100×(1−β)% bootstrapping confidence interval, or confidence set since only the integer values in the interval are relevant, in (13) provides a basic assessment of $K_{\alpha }$’s whereabouts. However its coverage does necessarily converge to the claimed value 1−β as *n* increasing indefinitely, due to the above mentioned possible inconsistency of ${\hat{K}}_{\alpha }$ and the consequential “at-most-one” over-estimation asymptotically. To take that into consideration, a conservative adjustment may be adopted by extending the lower limit of (13) by one, that is,

(15) $$[2{\hat{K}}_{\alpha }-{\hat{K}}_{\alpha ,1-\beta /2}^{*}-1,\;2{\hat{K}}_{\alpha }-{\hat{K}}_{\alpha ,\beta /2}^{*}].$$

An advantage of (15) is that its asymptotic coverage is at least 1−β for general $p_{↓}$, but a disadvantage is that the limiting form of (15) necessarily contains two integer values instead of one, which (13) could achieve when ${\hat{K}}_{\alpha }$ is consistent.

On the other hand, while (15) accommodates the issue of possible asymptotic over-estimation (by at most one) by ${\hat{K}}_{\alpha }$, in most practical cases, the more acute issue is still the under-estimation of $K_{\alpha }$ by ${\hat{K}}_{\alpha }$ when *n* is not sufficiently large. The confidence set in (15) generally requires *n* to be quite large for its coverage to be reasonably close to the claimed coverage 1−β. To help accelerate the convergence of the actual coverage to the claimed coverage, a more conservative adjustment may be adopted by extending the right limit of (15) by one, that is,

(16) $$[2{\hat{K}}_{\alpha }-{\hat{K}}_{\alpha ,1-\beta /2}^{*}-1,\;2{\hat{K}}_{\alpha }-{\hat{K}}_{\alpha ,\beta /2}^{*}+1].$$

Advantages of (16) are that its asymptotic coverage is at least 1−β for general $p_{↓}$ and that its actual coverage converges to at least 1−β faster as *n* increases. However a disadvantage is that the limiting form of (16) necessarily contains three integer values and no fewer.

The bootstrapping confidence intervals, described in (13), (15) and (16), may also be utilized in testing hypothesis with different degrees of conservativeness. For example, based on (13) and at the β level of significance, in testing $H_{0}:K_{\alpha }=k_{\alpha }$ versus $H_{a}:K_{\alpha }>k_{\alpha }$, $H_{a}:K_{\alpha }<k_{\alpha }$ or $H_{a}:K_{\alpha }\neq k_{\alpha }$, $k_{\alpha }$ is a pre-specified positive integer, one may choose to reject $H_{0}$ when

(17) $$k_{\alpha }<2{\hat{K}}_{\alpha }-{\hat{K}}_{\alpha ,1-\beta }^{*},$$

(18) $$k_{\alpha }>2{\hat{K}}_{\alpha }-{\hat{K}}_{\alpha ,\beta }^{*},\;\mathrm{or}$$

(19) $$k_{\alpha }\notin [2{\hat{K}}_{\alpha }-{\hat{K}}_{\alpha ,1-\beta /2}^{*},\;2{\hat{K}}_{\alpha }-{\hat{K}}_{\alpha ,\beta /2}^{*}]$$

respectively.

Based on (15) and at the β level of significance, in testing $H_{0}:K_{\alpha }=k_{\alpha }$ versus $H_{a}:K_{\alpha }>k_{\alpha }$, $H_{a}:K_{\alpha }<k_{\alpha }$ or $H_{a}:K_{\alpha }\neq k_{\alpha }$, $k_{\alpha }$ is a pre-specified positive integer, one may choose to reject $H_{0}$ when

(20) $$k_{\alpha }<2{\hat{K}}_{\alpha }-{\hat{K}}_{\alpha ,1-\beta }^{*}-1,$$

(21) $$k_{\alpha }>2{\hat{K}}_{\alpha }-{\hat{K}}_{\alpha ,\beta }^{*},\;\mathrm{or}$$

(22) $$k_{\alpha }\notin [2{\hat{K}}_{\alpha }-{\hat{K}}_{\alpha ,1-\beta /2}^{*}-1,\;2{\hat{K}}_{\alpha }-{\hat{K}}_{\alpha ,\beta /2}^{*}]$$

respectively.

Based on (16) and at the β level of significance, in testing $H_{0}:K_{\alpha }=k_{\alpha }$ versus $H_{a}:K_{\alpha }>k_{\alpha }$, $H_{a}:K_{\alpha }<k_{\alpha }$ or $H_{a}:K_{\alpha }\neq k_{\alpha }$, $k_{\alpha }$ is a pre-specified positive integer, one may choose to reject $H_{0}$ when

(23) $$k_{\alpha }<2{\hat{K}}_{\alpha }-{\hat{K}}_{\alpha ,1-\beta }^{*}-1,$$

(24) $$k_{\alpha }>2{\hat{K}}_{\alpha }-{\hat{K}}_{\alpha ,\beta }^{*}+1,\;\mathrm{or}$$

(25) $$k_{\alpha }\notin [2{\hat{K}}_{\alpha }-{\hat{K}}_{\alpha ,1-\beta /2}^{*}-1,\;2{\hat{K}}_{\alpha }-{\hat{K}}_{\alpha ,\beta /2}^{*}+1]$$

respectively.

Suppose there are two communities and it is of interest to estimate the difference between the two α-trimmed richness indices,

(26) $$D_{\alpha }=K_{1,\alpha }-K_{2,\alpha }$$

where $K_{1,\alpha }$ and $K_{2,\alpha }$ are α-trimmed richness indices of the two underlying communities, respectively. The proposed estimator of $D_{\alpha }$ in (26) is

(27) $${\hat{D}}_{\alpha }^{♯}={\hat{K}}_{1,\alpha }^{♯}-{\hat{K}}_{2,\alpha }^{♯}$$

where ${\hat{K}}_{1,\alpha }^{♯}=2{\hat{K}}_{1,\alpha }-{\hat{K}}_{1,\alpha ,1/2}^{*}$ and ${\hat{K}}_{2,\alpha }^{♯}=2{\hat{K}}_{2,\alpha }-{\hat{K}}_{2,\alpha ,1/2}^{*}$, where ${\hat{K}}_{1,\alpha }$ and ${\hat{K}}_{2,\alpha }$ are as in (7) and ${\hat{K}}_{1,\alpha ,1/2}^{*}$ and ${\hat{K}}_{2,\alpha ,1/2}^{*}$ are respective bootstrapping medians from the two samples as in (14).

In testing equality of generalized species richness of two communities, $D_{\alpha }=K_{1,\alpha }-K_{2,\alpha }$, one may first consider a bootstrapping 1−β confidence interval for $D_{\alpha }$ based on two independent samples are size $n_{1}$ and $n_{2}$, respectively,

(28) $$\left[2{\hat{D}}_{\alpha }-{\hat{D}}_{\alpha ,1-\beta /2}^{*},\;2{\hat{D}}_{\alpha }-{\hat{D}}_{\alpha ,\beta /2}^{*}\right]$$

where ${\hat{D}}_{\alpha }={\hat{K}}_{1,\alpha }-{\hat{K}}_{2,\alpha }$,

(29) $$\left[2{\hat{D}}_{\alpha }-{\hat{D}}_{\alpha ,1-\beta /2}^{*}-1,\;2{\hat{D}}_{\alpha }-{\hat{D}}_{\alpha ,\beta /2}^{*}+1\right]$$

where ${\hat{D}}_{\alpha ,\beta /2}^{*}$ and ${\hat{D}}_{\alpha ,1-\beta /2}^{*}$ are the 100×β/2 th and the 100×(1−β/2) th percentiles of the bootstrapping estimates, each of which is based a sample of size $n_{1}$ from ${\hat{p}}_{1,↓}$ and a sample of size $n_{2}$ from ${\hat{p}}_{2,↓}$, where, for j=1 or j=2, ${\hat{p}}_{j,↓}$ is the ordered relative frequencies of letters in the sample of size $n_{j}$ from the *j* th community.

For, $H_{0}:K_{1,\alpha }-K_{2,\alpha }=d_{0}$ versus $H_{1}:K_{1,\alpha }-K_{2,\alpha }>d_{0}$, or $H_{2}:K_{1,\alpha }-K_{2,\alpha }\neq d_{0}$, where $K_{\alpha ,1}$ and $K_{\alpha ,2}$ are the respective generalized species richness of two communities and $d_{0}$ is a pre-fixed integer, approximate testing procedures may be devised based (28) or (29). For example, based on (28), one may choose to reject $H_{0}$ when

(30) $$d_{0}<2{\hat{D}}_{\alpha }-{\hat{D}}_{\alpha ,1-\beta }^{*},\;\mathrm{for}H_{0}\mathrm{vs}.H_{1},\;\mathrm{or}$$

(31) $$d_{0}\notin \left[2{\hat{D}}_{\alpha }-{\hat{D}}_{\alpha ,1-\beta /2}^{*},\;2{\hat{D}}_{\alpha }-{\hat{D}}_{\alpha ,\beta /2}^{*}\right],\;\mathrm{for}H_{0}\mathrm{vs}.H_{2}.$$

Similarly, based on (29), one may choose to reject $H_{0}$ when

(32) $$d_{0}<2{\hat{D}}_{\alpha }-{\hat{D}}_{\alpha ,1-\beta }^{*}-1,\;\mathrm{for}H_{0}\mathrm{vs}.H_{1},\;\mathrm{or}$$

(33) $$d_{0}\notin \left[2{\hat{D}}_{\alpha }-{\hat{D}}_{\alpha ,1-\beta /2}^{*}-1,\;2{\hat{D}}_{\alpha }-{\hat{D}}_{\alpha ,\beta /2}^{*}+1\right],\;\mathrm{for}H_{0}\mathrm{vs}.H_{2}.$$

To assess the reliability of the inferential procedures discussed above, several simulation studies are conducted. The studies are carried out under three different distributions. The first distribution is the uniform distribution with K=20 and $p_{k}=0.05$ for k=1,⋯,20. The second distribution is a triangular distribution with K=20 and $p_{k}=k/20$ for k=1,⋯,20. The third distribution is the Poisson distribution with λ=10 and $p_{k}=e^{-\lambda }\lambda^{x}/x!$, noting that in this case *K* is infinite.

In Table 1, Table 2 and Table 3, the bias and the mean squared errors of ${\hat{K}}_{\alpha }$ of (7) and ${\hat{K}}_{\alpha }^{♯}$ of (14) are compared, at two levels of α, α=0.01 and α=0.05, for various sample sizes, *n*. Table 1, Table 2 and Table 3, respectively, summarize the results under three different distributions, the uniform, the triangular and the Poisson. Each simulation scenario is based on 5000 repeated samples. Each sample is bootstrapped 1000 times. The bias is defined in such a way that, a positive value indicates an under-estimation and a negative value indicates an over-estimation. The variable *T* is the average of Turing’s formula, $T_{n}=n_{1}/n$, where $n_{1}$ is the number of singletons in a sample, based on 5000 simulated samples. *T* helps to indicate the adequacy of sample size. Turing’s formula, $T_{n}$, is sometimes called the sample coverage deficit and $1-T_{n}$ is the sample coverage (see [17]).

It is quite clear that ${\hat{K}}_{\alpha }^{♯}$ generally has a smaller simulated bias than ${\hat{K}}_{\alpha }$. More specifically, if one considers an absolute bias being less than one to be satisfactory, then ${\hat{K}}_{\alpha }^{♯}$ gets there faster, as *n* increases, than ${\hat{K}}_{\alpha }$ in all cases considered in the simulation studies.

To assess the performance of the confidence sets in (13), (15) and (16), their actual coverage rates are evaluated by simulation studies with 1−β=0.95 for various sample sizes and distributions. For each scenario, the coverage rate is based on 5000 simulated samples and for each sample, the bootstrapping confidence set is based on 1000 bootstrapping samples. The results are summarized in Table 4, Table 5 and Table 6.

Let it be noted that, although the confidence set of (13) could perform well in some cases (see Columns 3 and 6 in Table 4, and Columns 6 and 12 of Table 5), it has difficulty in providing an appropriate coverage in many other cases (see Column 12 of Table 4, Columns 3, 6, 9 and 12 of Table 5, and Columns 3 and 9 of Table 6). The said difficulty is partially caused by the inconsistency mentioned above in combinations of certain distributional characteristics and the values of α. Similarly, the confidence set of (15) suffers from the same difficulty though to a lesser degree. It could also perform well in some cases (see Columns 4, 7, 10 and 13 in Table 4, Columns 7 and 10 of Table 5, and Columns 7 and 10 of Table 6), but it does not in many other cases (see Column 4 of Table 5, Columns 4 and 9 of Table 6). Since in practice the underlying distribution is not observable, it cannot be determined a priori what values of α are appropriate and what are not. This fact essentially disqualifies the confidence sets of (13) and (15) as general inferential procedures, but (16). Additionally, to be noted is the fact that the confidence set of (16) performs well across all cases in the simulation studies albeit more conservative. The confidence sets of (28) and (29) have general better performances than their one-sample counterparts due to an offset of bias between the two one-sample estimators.

Another point of interest pertains to the practically important question of how large a sample should be in order for (16) to produce a reasonable coverage. Simulation results in Table 4, Table 5 and Table 6 seem to indicate that the coverage is adequate when Turing’s formula, which estimates the total probability associated with the letters of the alphabet not represented in a given sample, takes on a value approximately at a level not much greater than α, that is, $T=n_{1}/n<\alpha$ where $n_{1}$ is the number of species observed exactly once in the sample, referred to as the rule of thumb below. (Interested readers may refer to Zhang (2017) for a comprehensive introduction to Turing’s formula.)

In summary, all things considered, observing the rule of thumb,

(14) is the proposed estimator of $K_{\alpha }$;
(16) is the proposed 100×(1−β)% confidence set for $K_{\alpha }$;
(23)–(25) are the proposed approximate size-β tests of hypothesis involving $K_{\alpha }$;
(29) is the proposed 100×(1−β)% confidence set for $D_{\alpha }=K_{1,\alpha }-K_{2,\alpha }$; and
(32) and (33) are the proposed approximate size-β tests of hypothesis involving $D_{\alpha }$.

**Example** **8.** *Two tree samples of 1-ha plots (#6 and #18), respectively, indexed as samples 6 and 18, of tropical forest in the experimental forest of Paracou, French Guiana, described in [22], are compared in terms of biodiversity. Respectively 643 and 481 trees with diameter at breast height over 10 cm were inventoried. The data is available in the entropart package for R. In these samples, 147 and 149 tree species from plots #6 and #18 are, respectively, observed, along with their frequencies. In [23], the data are analyzed by using generalized Simpson’s indices and concluded that plot #18 is more diverse than plot #6. In the respective samples, Turing’s formula takes on the values of $T_{6}=10.58\%$ and $T_{18}=15.38\%$. Observing the rule of thumb, let the generalized species richness be evaluated at α=0.15. ${\hat{K}}_{6,0.15}^{♯}=76$, ${\hat{K}}_{18,0.15}^{♯}=91$ (as compared to the plug-ins ${\hat{K}}_{6,0.15}=65$ and ${\hat{K}}_{18,0.15}=77$), and therefore $\hat{D}={\hat{K}}_{18,0.15}^{♯}-{\hat{K}}_{6,0.15}^{♯}=15$. The proposed 95% confidence sets for $K_{6,0.15}$ and $K_{18,0.15}$ are, respectively, [69,82] and [84,98]. The proposed one-sided and two-sided 95% confidence sets for $D_{0.15}$ are, respectively, [6,∞) and [5,26], both of which exclude zero and therefore lead to a rejection of $H_{0}:K_{6,0.15}=K_{18,0.15}$ with either $H_{a}:K_{6,0.15}<K_{18,0.15}$ or $H_{a}:K_{6,0.15}\neq K_{18,0.15}$, qualitatively supporting the findings of [23].*

**Figure 2:**
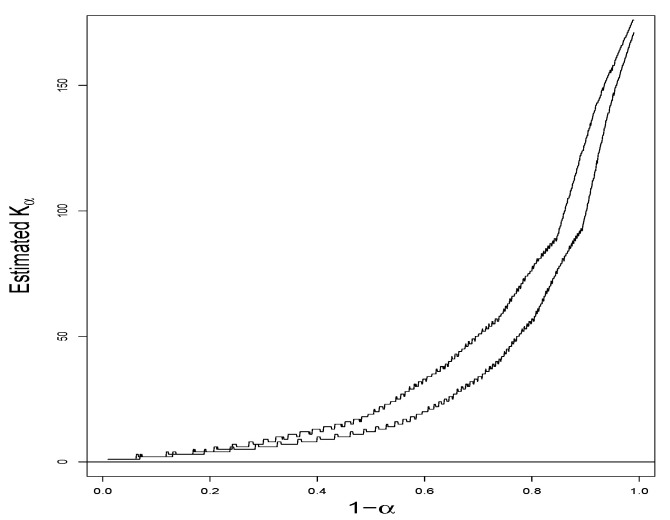
Estimated $K_{\alpha }$ for Plots #6 and #18.

**Figure 3:**
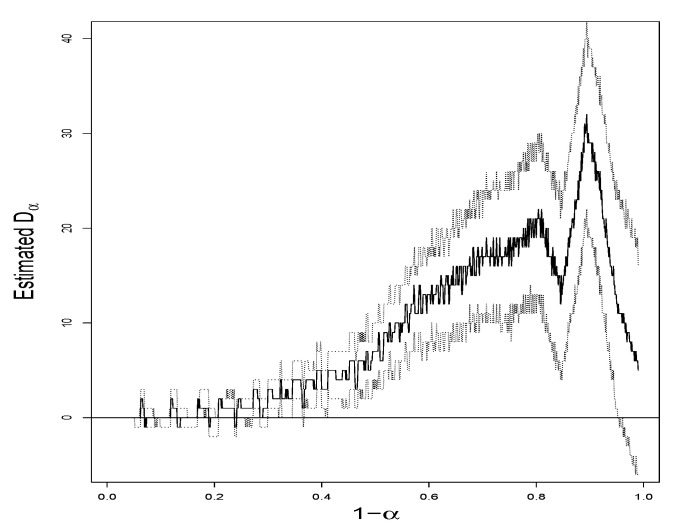
Estimated $D_{\alpha }$ with 95% Confidence Band.

*Let α vary from 0.01 to 0.99. ${\hat{K}}_{6,\alpha }^{♯}$ and ${\hat{K}}_{18,\alpha }^{♯}$ as functions of α by means of (14) give two curves in Figure 2, which visually suggests that plot #18 is more diverse than plot #6 for a wide range of α. ${\hat{D}}_{\alpha }={\hat{K}}_{18,\alpha }^{♯}-{\hat{K}}_{6,\alpha }^{♯}$ as a function of α, along with the 95% point-wise confidence band by means of (29), is given in Figure 3, where it is evident that, with reasonable statistical confidence, $K_{\alpha ,18}>K_{\alpha ,6}$ for α values in the range from 0.6 to 0.15, that is, for 1−α values from 0.4 to 0.85.*

## 4. Summary

This article proposes a generalized richness index, $K_{\alpha }$ of (3), or equivalently of (4) or of (5), and an estimator, ${\hat{K}}_{\alpha }^{♯}$ of (14). α∈[0,0) is a user-chosen constant, and when α=0, $K_{\alpha }$ becomes the well-known original richness index, *K*. $K_{\alpha }$ may also be referred to as the α-trimmed richness index. It is designed to remove or to alleviate several weaknesses of *K*. First, *K* is only finitely defined for some distributions but not for all. On the other hand, $K_{\alpha }$ is finitely defined for all distributions on a countable alphabet. Second, *K* does not take the abundance $\left\{p_{k};k\geq 1\right\}$ into consideration, but $K_{\alpha }$ does. Third, *K* is ultra-sensitive to re-distribution of an arbitrarily small mass, but $K_{\alpha }$ is not, as evidenced by Definitions 1 and 2, Examples 1 and 4, and Proposition A2.

A conservative confidence interval based on bootstrapping is proposed in (16). This confidence interval provides the basic support for inferences about $K_{\alpha }$. A rule of thumb to judge whether the sample is adequate in supporting the proposed methodology is also proposed based on Turing’s formula: $T=n_{1}/n<\alpha$, where $n_{1}$ is the number of singletons in the sample of size *n*. The rule of thumb is illustrated by simulated results in Table 4, Table 5 and Table 6. More specifically, in Table 4, the rule of thumb amounts to n≥110 for α=0.01, n≥60 for α=0.05, n≥50 for α=0.10 and n≥40 for α=0.15. The simulated coverages are all near or above the target 95%. In Table 5, the rule of thumb amounts to n≥150 for α=0.01, n≥70 for α=0.05, n≥50 for α=0.10 and n≥40 for α=0.15. The simulated coverages are all above the target 95%. In Table 6, the rule of thumb amounts to n≥450 for α=0.01, n≥70 for α=0.05, n≥40 for α=0.10 and n≥30 for α=0.15. The simulated coverages are all above the target 95%.

The one-sample estimator of $K_{\alpha }$ in (14) for a single community is extended to the two-sample estimator of $D_{\alpha }$ of (26), the difference of two α-trimmed richness indices of two communities. The proposed estimator of $D_{\alpha }$ is ${\hat{D}}_{\alpha }^{♯}$ as in (27). A proposed 100×(1−β)% confidence interval for $D_{\alpha }$ is given in (29). This interval provides the basic support for testing hypotheses regarding $D_{\alpha }$, as specified in (32) and (33).

For the two-sample problem, the rule of thumb for the one-sample problem is modified to be:

$$T_{1}=n_{1,1}/n_{1}<\alpha \;\mathrm{and}\;T_{2}=n_{2,1}/n_{2}<\alpha$$

where $n_{1}$ and $n_{2}$ are the respective sample sizes of the two independent samples, and $n_{1,1}$ and $n_{2,1}$ are the respective numbers of singletons in the two independent samples.

## Figures and Tables

**Table 1:** Simulation Results under Uniform Distribution, K=20.

*n*	T	α=0.01	${\hat{K}}_{\alpha }$ Bias	${\hat{K}}_{\alpha }$ MSE	${\hat{K}}_{\alpha }^{♯}$ Bias	${\hat{K}}_{\alpha }^{♯}$ MSE	α=0.05	${\hat{K}}_{\alpha }$ Bias	${\hat{K}}_{\alpha }$ MSE	${\hat{K}}_{\alpha }^{♯}$ Bias	${\hat{K}}_{\alpha }^{♯}$ MSE
10	0.63	$K_{\alpha }=20$	12.00	145.21	9.72	97.20	$K_{\alpha }=19$	11.00	122.20	8.72	78.76
20	0.38	$K_{\alpha }=20$	7.17	53.32	3.96	20.17	$K_{\alpha }=19$	7.17	53.32	3.97	20.21
30	0.22	$K_{\alpha }=20$	4.33	20.76	1.16	5.70	$K_{\alpha }=19$	4.33	20.76	1.17	5.71
40	0.14	$K_{\alpha }=20$	2.57	8.18	−0.26	3.25	$K_{\alpha }=19$	3.56	14.23	0.76	3.76
50	0.08	$K_{\alpha }=20$	1.53	3.46	−0.81	2.85	$K_{\alpha }=19$	2.48	7.25	0.17	2.13
60	0.05	$K_{\alpha }=20$	0.94	1.66	−0.92	2.32	$K_{\alpha }=19$	2.52	7.08	0.55	1.74
70	0.03	$K_{\alpha }=20$	0.55	0.77	−0.87	1.73	$K_{\alpha }=19$	1.79	3.87	0.06	1.40
80	0.02	$K_{\alpha }=20$	0.33	0.41	−0.73	1.07	$K_{\alpha }=19$	1.75	3.59	0.19	1.24
90	0.01	$K_{\alpha }=20$	0.19	0.22	−0.62	0.83	$K_{\alpha }=19$	1.33	2.18	−0.01	0.90
100	0.01	$K_{\alpha }=20$	0.61	0.71	−0.25	0.76	$K_{\alpha }=19$	1.37	2.28	0.15	0.89
110	0.00	$K_{\alpha }=20$	0.41	0.45	−0.35	0.91	$K_{\alpha }=19$	1.04	1.41	−0.09	0.82
120	0.00	$K_{\alpha }=20$	0.28	0.30	−0.49	0.95	$K_{\alpha }=19$	1.12	1.52	0.08	0.64

**Table 2:** Simulation Results under Triangular Distribution, K=20.

*n*	T	α=0.01	${\hat{K}}_{\alpha }$ Bias	${\hat{K}}_{\alpha }$ MSE	${\hat{K}}_{\alpha }^{♯}$ Bias	${\hat{K}}_{\alpha }^{♯}$ MSE	α=0.05	${\hat{K}}_{\alpha }$ Bias	${\hat{K}}_{\alpha }$ MSE	${\hat{K}}_{\alpha }^{♯}$ Bias	${\hat{K}}_{\alpha }^{♯}$ MSE
10	0.55	$K_{\alpha }=19$	11.45	132.23	9.39	91.09	$K_{\alpha }=16$	8.44	72.55	6.39	43.72
20	0.31	$K_{\alpha }=19$	7.34	55.85	4.66	26.22	$K_{\alpha }=16$	5.34	30.50	2.67	11.64
30	0.18	$K_{\alpha }=19$	5.01	27.24	2.42	10.23	$K_{\alpha }=16$	3.01	11.18	0.43	4.57
40	0.12	$K_{\alpha }=19$	3.55	14.51	1.22	5.24	$K_{\alpha }=16$	2.52	8.19	0.26	3.80
50	0.08	$K_{\alpha }=19$	2.61	8.51	0.57	3.52	$K_{\alpha }=16$	1.55	3.97	−0.43	3.24
60	0.06	$K_{\alpha }=19$	1.95	5.35	0.15	2.94	$K_{\alpha }=16$	1.64	3.92	−0.01	2.35
70	0.04	$K_{\alpha }=19$	1.48	3.52	−0.08	2.47	$K_{\alpha }=16$	1.04	2.19	−0.40	2.26
80	0.03	$K_{\alpha }=19$	1.14	2.49	−0.23	2.20	$K_{\alpha }=16$	1.13	2.27	−0.13	1.85
90	0.03	$K_{\alpha }=19$	0.84	1.76	−0.38	2.00	$K_{\alpha }=16$	0.69	1.37	−0.45	1.82
100	0.02	$K_{\alpha }=19$	1.55	3.33	0.50	1.89	$K_{\alpha }=16$	0.80	1.48	−0.20	1.53
110	0.02	$K_{\alpha }=19$	1.33	2.57	0.37	1.57	$K_{\alpha }=16$	0.48	0.96	−0.45	1.52
120	0.01	$K_{\alpha }=19$	1.15	2.04	0.29	1.33	$K_{\alpha }=16$	0.56	1.01	−0.30	1.34

**Table 3:** Simulation Results under Poisson Distribution, λ=10.

*n*	T	α=0.01	${\hat{K}}_{\alpha }$ Bias	${\hat{K}}_{\alpha }$ MSE	${\hat{K}}_{\alpha }^{♯}$ Bias	${\hat{K}}_{\alpha }^{♯}$ MSE	α=0.05	${\hat{K}}_{\alpha }$ Bias	${\hat{K}}_{\alpha }$ MSE	${\hat{K}}_{\alpha }^{♯}$ Bias	${\hat{K}}_{\alpha }^{♯}$ MSE
10	0.46	$K_{\alpha }=16$	9.10	84.00	7.38	57.25	$K_{\alpha }=13$	6.10	38.42	4.38	22.00
20	0.22	$K_{\alpha }=16$	6.01	37.97	4.05	20.26	$K_{\alpha }=13$	4.01	17.86	2.08	8.20
30	0.13	$K_{\alpha }=16$	4.39	21.17	2.59	10.48	$K_{\alpha }=13$	3.08	7.52	0.61	4.17
40	0.09	$K_{\alpha }=16$	3.36	13.16	1.76	6.65	$K_{\alpha }=13$	2.25	6.67	0.79	3.89
50	0.06	$K_{\alpha }=16$	2.64	8.70	1.16	4.62	$K_{\alpha }=13$	1.50	3.75	0.19	2.98
60	0.05	$K_{\alpha }=16$	2.08	6.06	0.69	3.68	$K_{\alpha }=13$	1.65	3.94	0.57	2.65
70	0.04	$K_{\alpha }=16$	1.62	4.32	0.29	3.21	$K_{\alpha }=13$	1.13	2.52	0.15	2.35
80	0.03	$K_{\alpha }=16$	1.26	3.21	0.01	2.88	$K_{\alpha }=13$	1.31	2.74	0.49	2.16
90	0.03	$K_{\alpha }=16$	0.94	2.54	−0.28	3.08	$K_{\alpha }=13$	0.94	1.86	0.16	1.89
100	0.03	$K_{\alpha }=16$	1.59	4.06	0.49	3.12	$K_{\alpha }=13$	1.07	2.02	0.39	1.83
110	0.02	$K_{\alpha }=16$	1.37	3.39	0.32	2.92	$K_{\alpha }=13$	0.82	1.54	0.19	1.69
120	0.02	$K_{\alpha }=16$	1.17	2.81	0.15	2.77	$K_{\alpha }=13$	0.95	1.68	0.38	1.65

**Table 4:** Simulated Coverage of 95% Confidence Sets under Uniform Distribution, K=20.

		α=0.01			α=0.05			α=0.10			α=0.15		
*n*	T	Of (13)	Of (15)	Of (16)	Of (13)	Of (15)	Of (16)	Of (13)	Of (15)	Of (16)	Of (13)	Of (15)	Of (16)
10	0.63	0.0000	0.0000	0.0000	0.0000	0.0000	0.0000	0.0000	0.0000	0.0000	0.0000	0.0000	0.0000
20	0.38	0.3226	0.3226	0.5284	0.3224	0.3224	0.5060	0.3222	0.3222	0.4118	0.6332	0.6332	0.6774
30	0.22	0.7898	0.8056	0.9086	0.7874	0.8032	0.9010	0.5636	0.5642	0.7704	0.8084	0.8090	0.9128
40	0.14	0.9124	0.9422	0.9794	0.8522	0.8584	0.9420	0.6576	0.6576	0.8402	0.9534	0.9532	0.9732
50	0.08	0.8884	0.9862	0.9964	0.9588	0.9596	0.9964	0.7868	0.7922	0.9382	0.9622	0.9676	0.9958
60	0.05	0.9792	0.9934	0.9988	0.9302	0.9318	0.9858	0.8678	0.8682	0.9488	0.9910	0.9918	0.9998
70	0.03	0.9970	0.9970	0.9996	0.9512	0.9824	0.9986	0.8586	0.8586	0.9820	0.9984	0.9984	0.9986
80	0.02	0.9990	0.9990	1.0000	0.9770	0.9834	0.9954	0.9228	0.9228	0.9882	0.9992	0.9994	1.0000
90	0.01	0.9992	0.9992	1.0000	0.9360	0.9816	0.9984	0.9552	0.9554	0.9848	0.9988	0.9990	1.0000
100	0.01	0.9940	0.9982	1.0000	0.9284	0.9762	0.9992	0.9322	0.9328	0.9886	0.9990	0.9990	0.9998
110	0.00	0.9964	0.9966	1.0000	0.8912	0.9952	0.9998	0.9316	0.9384	0.9970	0.9998	0.9998	1.0000
120	0.00	0.9958	0.9958	1.0000	0.9148	0.9942	1.0000	0.9282	0.9322	0.9990	0.9994	1.0000	1.0000
130	0.00	0.9984	0.9984	0.9998	0.8952	0.9966	1.0000	0.9520	0.9596	0.9992	0.9984	1.0000	1.0000
140	0.00	0.9990	0.9990	1.0000	0.8918	0.9916	0.9994	0.9662	0.9782	0.9998	0.9992	1.0000	1.0000
150	0.00	0.9998	0.9998	1.0000	0.9594	0.9918	1.0000	0.9764	0.9874	0.9990	0.9968	1.0000	1.0000
200	0.00	0.9966	0.9966	1.0000	0.9886	0.9960	1.0000	0.8846	0.9414	1.0000	0.9916	1.0000	1.0000
250	0.00	0.9974	0.9974	1.0000	0.9996	0.9996	1.0000	0.8608	0.9750	0.9998	0.9642	1.0000	1.0000
300	0.00	0.9964	0.9964	1.0000	0.9992	0.9992	1.0000	0.9120	0.9948	1.0000	0.9374	1.0000	1.0000
350	0.00	0.9996	0.9996	1.0000	1.0000	1.0000	1.0000	0.9770	0.9982	1.0000	0.8744	1.0000	1.0000
400	0.00	0.9998	0.9998	1.0000	1.0000	1.0000	1.0000	0.9962	1.0000	1.0000	0.8696	1.0000	1.0000
450	0.00	1.0000	1.0000	1.0000	1.0000	1.0000	1.0000	0.9904	0.9916	1.0000	0.9216	1.0000	1.0000
500	0.00	1.0000	1.0000	1.0000	0.9938	0.9938	1.0000	0.9574	0.9574	1.0000	0.9466	1.0000	1.0000
1000	0.00	1.0000	1.0000	1.0000	1.0000	1.0000	1.0000	0.9988	0.9988	1.0000	1.0000	1.0000	1.0000

**Table 5:** Simulated Coverage of 95% Confidence Sets under Triangular Distribution, K=20.

		α=0.01			α=0.05			α=0.10			α=0.15		
*n*	T	Of (13)	Of (15)	Of (16)	Of (13)	Of (15)	Of (16)	Of (13)	Of (15)	Of (16)	Of (13)	Of (15)	Of (16)
10	0.55	0.0000	0.0000	0.0000	0.0024	0.0024	0.0254	0.0262	0.0262	0.1856	0.1914	0.1914	0.1938
20	0.31	0.2	0.2370	0.3026	0.5408	0.5444	0.6492	0.5466	0.5648	0.7866	0.5262	0.5444	0.7420
30	0.18	0.6074	0.6120	0.7400	0.8138	0.8378	0.9240	0.7802	0.8034	0.8692	0.7190	0.7426	0.8940
40	0.12	0.7718	0.7798	0.9052	0.8192	0.8700	0.9344	0.8016	0.8552	0.9602	0.7966	0.8156	0.9548
50	0.08	0.8548	0.8828	0.9440	0.8546	0.9188	0.9516	0.8864	0.9304	0.9650	0.8824	0.9184	0.9584
60	0.06	0.8848	0.9078	0.9720	0.9086	0.9426	0.9742	0.9052	0.9386	0.9740	0.8716	0.8928	0.9686
70	0.04	0.9144	0.9436	0.9752	0.8948	0.9494	0.9790	0.8900	0.9352	0.9812	0.8994	0.9152	0.9840
80	0.03	0.9004	0.9528	0.9876	0.9080	0.9416	0.9866	0.9058	0.9548	0.9872	0.9000	0.9158	0.9900
90	0.03	0.8830	0.9634	0.9922	0.8908	0.9682	0.9884	0.9150	0.9742	0.9922	0.9378	0.9636	0.9920
100	0.02	0.8916	0.8932	0.9768	0.9102	0.9660	0.9930	0.9082	0.9784	0.9916	0.9408	0.9648	0.9920
110	0.02	0.9174	0.9200	0.9862	0.8852	0.9834	0.9918	0.8806	0.9824	0.9932	0.9446	0.9788	0.9950
120	0.01	0.9384	0.9434	0.9868	0.9056	0.9796	0.9954	0.8818	0.9820	0.9938	0.9390	0.9662	0.9916
130	0.01	0.9422	0.9492	0.9874	0.8602	0.9798	0.9922	0.8670	0.9788	0.9952	0.9144	0.9580	0.9956
140	0.01	0.9334	0.9472	0.9894	0.8854	0.9722	0.9946	0.8418	0.9702	0.9930	0.9062	0.9426	0.9958
150	0.01	0.9264	0.9506	0.9918	0.8362	0.9760	0.9946	0.8410	0.9752	0.9956	0.8966	0.9482	0.9966
200	0.00	0.9026	0.9088	0.9928	0.8620	0.9808	0.9988	0.8320	0.9858	0.9964	0.9152	0.9610	0.9996
250	0.00	0.9348	0.9620	0.9976	0.8162	0.9942	0.9984	0.8098	0.9954	0.9974	0.9318	0.9868	1.0000
300	0.00	0.9504	0.9570	0.9962	0.8314	0.9946	0.9986	0.7910	0.9966	0.9978	0.9404	0.9928	0.9992
350	0.00	0.9502	0.9778	0.9972	0.7822	0.9932	0.9974	0.7664	0.9938	0.9968	0.9242	0.9938	0.9994
400	0.00	0.9558	0.9626	0.9934	0.8012	0.9888	0.9990	0.7406	0.9914	0.9994	0.9394	0.9830	0.9986
450	0.00	0.9334	0.9544	0.9972	0.7588	0.9858	0.9992	0.7286	0.9904	0.9984	0.8994	0.9588	0.9998
500	0.00	0.9166	0.9256	0.9976	0.7716	0.9848	0.9998	0.7060	0.9878	0.9992	0.8914	0.9376	1.0000
1000	0.00	0.8572	0.8592	0.9998	0.7432	0.9968	1.0000	0.6166	0.9978	1.0000	0.9290	0.9534	1.0000

**Table 6:** Simulated Coverage of 95% Confidence Sets under Poisson Distribution, λ=10.

		α=0.01			α=0.05			α=0.10			α=0.15		
*n*	T	Of (13)	Of (15)	Of (16)	Of (13)	Of (15)	Of (16)	Of (13)	Of (15)	Of (16)	Of (13)	Of (15)	Of (16)
10	0.46	0.0004	0.0004	0.0054	0.0736	0.0736	0.2998	0.3010	0.3010	0.3038	0.3224	0.3276	0.6372
20	0.22	0.2712	0.2714	0.3682	0.8644	0.8658	0.9306	0.6286	0.6542	0.8560	0.8608	0.8864	0.9588
30	0.13	0.5186	0.5214	0.6840	0.9002	0.9398	0.9716	0.7550	0.7904	0.8706	0.8994	0.9276	0.9666
40	0.09	0.6298	0.6400	0.8094	0.9632	0.9822	0.9872	0.7996	0.8166	0.9588	0.9648	0.9742	0.9954
50	0.06	0.7428	0.7534	0.8836	0.9446	0.9800	0.9816	0.8510	0.8774	0.9780	0.9640	0.9864	0.9966
60	0.05	0.8054	0.8242	0.9076	0.9718	0.9938	0.9944	0.8968	0.9206	0.9698	0.9858	0.9960	0.9984
70	0.04	0.8034	0.8500	0.9360	0.9320	0.9898	0.9902	0.8818	0.9206	0.9682	0.9742	0.9980	0.9992
80	0.03	0.8126	0.8768	0.9536	0.9720	0.9966	0.9966	0.8776	0.9082	0.9794	0.9874	0.9992	0.9996
90	0.03	0.7902	0.8840	0.9468	0.9486	0.9922	0.9922	0.8602	0.8904	0.9838	0.9772	0.9976	0.9994
100	0.03	0.7660	0.8052	0.9342	0.9732	0.9954	0.9954	0.8556	0.8830	0.9920	0.9868	0.9990	0.9998
110	0.02	0.7828	0.8342	0.9404	0.9588	0.9956	0.9956	0.8714	0.9040	0.9942	0.9826	0.9984	0.9998
120	0.02	0.7910	0.8592	0.9458	0.9736	0.9980	0.9980	0.8762	0.9022	0.9962	0.9892	0.9988	0.9998
130	0.02	0.7966	0.8876	0.9514	0.9622	0.9970	0.9970	0.8886	0.9142	0.9964	0.9838	0.9988	1.0000
140	0.02	0.7794	0.8966	0.9462	0.9730	0.9990	0.9990	0.9002	0.9296	0.9970	0.9880	0.9990	0.9998
150	0.02	0.7476	0.8894	0.9316	0.9672	0.9976	0.9976	0.9052	0.9316	0.9970	0.9854	0.9988	1.0000
200	0.01	0.8028	0.8870	0.9550	0.9800	0.9992	0.9992	0.9456	0.9696	0.9948	0.9920	1.0000	1.0000
250	0.01	0.7648	0.8948	0.9460	0.9734	0.9994	0.9994	0.9422	0.9720	0.9930	0.9930	1.0000	1.0000
300	0.01	0.8488	0.9178	0.9756	0.9780	0.9992	0.9992	0.9264	0.9546	0.9938	0.9974	1.0000	1.0000
350	0.01	0.8184	0.9294	0.9602	0.9650	0.9994	0.9994	0.8968	0.9242	0.9976	0.9962	1.0000	1.0000
400	0.01	0.8680	0.9486	0.9822	0.9794	0.9998	0.9998	0.8702	0.8944	0.9988	0.9972	1.0000	1.0000
450	0.00	0.8412	0.9542	0.9708	0.9752	0.9998	0.9998	0.8422	0.8618	0.9978	0.9982	1.0000	1.0000
500	0.00	0.8848	0.9656	0.9808	0.9776	1.0000	1.0000	0.8194	0.8382	0.9996	0.9994	1.0000	1.0000
1000	0.00	0.8656	0.9742	0.9890	0.9876	1.0000	1.0000	0.8578	0.8612	1.0000	1.0000	1.0000	1.0000

## Data Availability

The data used in Example 8 are available in the entropart package for R.

## Appendix A

The claims that $K_{\alpha }$ satisfies Axiom $A_{4}$ and that $K_{\alpha }$ is 100×α% robust (the claim of Example 2) are established in this section.

For clarity of the proof, a definition and two lemmas are needed. The generalized species richness $K_{\alpha }\left(p_{↓}\right)$ as in (3), (4) or (5) is defined for an underlying p being a probability distribution, that is, more specifically $p_{k}\geq 0$ for each *k* and $\sum_{k\geq 1}p_{k}=1$. For notation convenience in the proofs of this section, let the definition of $K_{\alpha }\left(p_{↓}\right)$ be extended to any sequence of non-negative numbers, $p=\{p_{k};k\geq 1\}$ or $p_{↓}=\left\{p_{\left(k\right)};k\geq 1\right\}$, such that $\sum_{k\geq 1}p_{k}<\infty$, which implies that $p_{\left(k\right)}\to 0$ as k→∞, specifically noting that $\sum_{k\geq 1}p_{k}$ may not necessarily be one.

**Definition** **A1.**
*For any sequence of non-negative values $p=\{p_{k};k\geq 1\}$, such that $\sum_{k\geq 1}p_{k}<\infty$, and an $\alpha \in (0,\sum_{k\geq 1}p_{k})$, the generalized species richness is given by*

(A1)
$$K_{\alpha }=K_{\alpha }\left(p_{↓}\right)=\min\left\{k:\sum_{i=k}^{\infty }p_{\left(i\right)}<\alpha \right\}-1.$$

It is clear that if p is a bonafide probability distribution, then $K_{\alpha }\left(p_{↓}\right)$ given in (3), (4) or (5) is identical to (A1) in Definition A1. In this section, the notion of $K_{\alpha }$ used is that of (A1).

**Lemma** **A1.**
*For any given sequence of non-negative values $p=\{p_{k};k\geq 1\}$, let a mass of ε>0 be taken away from $p_{i}$ for a specific index i, where $\varepsilon \in (0,p_{i}]$. Let $p_{i}^{*}=p_{i}-\varepsilon$, let $p^{*}$ be the sequence p but with $p_{i}^{*}$ in place of $p_{i}$, and let $p_{↓}^{*}=\left\{p_{\left(k\right)}^{*};k\geq 1\right\}$ be the re-arranged $p^{*}$ in a non-increasing order. For any $\alpha \in (0,\sum_{k\geq 1}p_{k})$, $K_{\alpha }\left(p_{↓}^{*}\right)\leq K_{\alpha }\left(p_{↓}\right)$.*

**Proof.** Without loss of generality, let it be assumed that the sequence $p=\{p_{k};k\geq 1\}$ is non-increasingly arranged. Denote $K_{\alpha }\left(p_{↓}\right)=k_{\alpha }$ and note that

(A2) $$\sum_{k=k_{\alpha }}^{\infty }p_{\left(k\right)}\geq \alpha \;\mathrm{and}\;\sum_{k=k_{\alpha }+1}^{\infty }p_{\left(k\right)}<\alpha .$$

The lemma is established, respectively, in three exhaustive scenarios: (a) $i<k_{\alpha }$, (b) $i=k_{\alpha }$, and $i>k_{\alpha }$. In scenario (a), there are three exhaustive possible placements of $p_{i}^{*}$ in $p_{↓}^{*}$ and they are

(a1):	$p_{i}^{*}<p_{k_{\alpha }}$,	$p_{↓}^{*}=\left\{p_{1},\cdots ,p_{i}^{*},\cdots ,p_{k_{\alpha }},\cdots \right\}$,
(a2):	$p_{i}^{*}=p_{k_{\alpha }}$,	$p_{↓}^{*}=\left\{p_{1},\cdots ,p_{i}^{*},p_{k_{\alpha }},\cdots \right\}$,
(a3):	$p_{i}^{*}>p_{k_{\alpha }}$,	$p_{↓}^{*}=\left\{p_{1},\cdots ,p_{k_{\alpha }},\cdots ,p_{i}^{*},\cdots \right\}$.

In either scenario (a1) or scenario (a2), the right tail sub-sequence $\left\{p_{k_{\alpha }},\cdots \right\}$ of p is preserved in $p_{↓}^{*}$, and therefore $K_{\alpha }\left(p_{↓}^{*}\right)=K_{\alpha }\left(p_{↓}\right)$. In scenario (a3), $p_{k_{\alpha }}$ occupies the $k_{\alpha }-1$ st position in $p_{↓}^{*}$. It follows that

(A3) $$\tau^{*}\left(k_{\alpha }\right)=\sum_{k\geq k_{\alpha }}p_{\left(k\right)}^{*}=p_{i}^{*}+\sum_{k\geq k_{\alpha }+1}p_{k}.$$

Noting $\sum_{k\geq k_{\alpha }+1}p_{k}<\alpha$ by (A2), if $\tau^{*}\left(k_{\alpha }\right)\geq \alpha$ then $K_{\alpha }\left(p_{↓}^{*}\right)=k_{\alpha }=K_{\alpha }\left(p_{↓}\right)$. If $\tau^{*}\left(k_{\alpha }\right)<\alpha$ then, again by (A2), $K_{\alpha }\left(p_{↓}^{*}\right)=k_{\alpha }-1<K_{\alpha }\left(p_{↓}\right)$. In scenario (b), (A3) still holds. Noting $\sum_{k\geq k_{\alpha }+1}p_{k}<\alpha$, if $\tau^{*}\left(k_{\alpha }\right)\geq \alpha$ then $K_{\alpha }\left(p_{↓}^{*}\right)=k_{\alpha }=K_{\alpha }\left(p_{↓}\right)$. If $\tau^{*}\left(k_{\alpha }\right)<\alpha$ then, since $p_{k_{\alpha }-1}\geq p_{k_{\alpha }}$, $K_{\alpha }\left(p_{↓}^{*}\right)=k_{\alpha }-1<K_{\alpha }\left(p_{↓}\right)$. In scenario (c), it follows that $p_{k_{\alpha }}$ occupies the $k_{\alpha }$ th position in $p_{↓}^{*}$ and that

(A4) $$\tau^{*}\left(k_{\alpha }+1\right)=\sum_{k\geq k_{\alpha }+1}p_{\left(k\right)}^{*}=\sum_{k\geq k_{\alpha }+1}p_{k}-\left(p_{i}-p_{i}^{*}\right)<\alpha .$$

If $\tau^{*}\left(k_{\alpha }\right)=\sum_{k\geq k_{\alpha }}p_{k}-\left(p_{i}-p_{i}^{*}\right)\geq \alpha$ then $K_{\alpha }\left(p_{↓}^{*}\right)=k_{\alpha }=K_{\alpha }\left(p_{↓}\right)$. If $\tau^{*}\left(k_{\alpha }\right)<\alpha$ then

$$\begin{array}{cc} \tau^{*}\left(k_{\alpha }-1\right) & =p_{k_{\alpha }-1}+\tau^{*}\left(k_{\alpha }\right)=p_{k_{\alpha }-1}+\sum_{k\geq k_{\alpha }}p_{k}-\left(p_{i}-p_{i}^{*}\right)\\ \; & =\sum_{k\geq k_{\alpha }}p_{k}+\left(p_{k_{\alpha }-1}-p_{i}\right)+p_{i}^{*}>\sum_{k\geq k_{\alpha }}p_{k}\geq \alpha . \end{array}$$

It follows that $K_{\alpha }\left(p_{↓}^{*}\right)=k_{\alpha }-1<K_{\alpha }\left(p_{↓}\right)$. □

The proof of Lemma A1 above actually establishes that $K_{\alpha }\left(p_{↓}\right)-1\leq K_{\alpha }\left(p_{↓}^{*}\right)\leq K_{\alpha }\left(p_{↓}\right)$, which immediately gives the following corollary.

**Corollary** **A1.**
*For any given sequence of non-negative values $p=\{p_{k};k\geq 1\}$, let a mass of ε>0 be added to $p_{i}$ for a specific index i. Let $p_{i}^{*}=p_{i}+\varepsilon$, let $p^{*}$ be the sequence p but with $p_{i}^{*}$ in place of $p_{i}$, and let $p_{↓}^{*}=\left\{p_{\left(k\right)}^{*};k\geq 1\right\}$ be the re-arranged $p^{*}$ in a non-increasing order. For any $\alpha \in (0,\sum_{k\geq 1}p_{k})$, $K_{\alpha }\left(p_{↓}\right)\leq K_{\alpha }\left(p_{↓}^{*}\right)\leq K_{\alpha }\left(p_{↓}\right)+1$.*

**Lemma** **A2.**
*For any given sequence of non-negative values $p=\{p_{k};k\geq 1\}$ and a given $\alpha \in (0,\sum_{k\geq 1}p_{k})$, let a mass of ε>0 be added to either $p_{i}$ or $p_{j}$, where i and j are two specific indices such that $p_{i}>p_{j}$, resulting in $p_{i}^{*}=p_{i}+\varepsilon$ and $p_{j}^{*}=p_{j}+\varepsilon$. Let $p^{*}\left(i\right)$ be the sequence p but with $p_{i}^{*}$ in place of $p_{i}$. Let $p^{*}\left(j\right)$ be the sequence p but with $p_{j}^{*}$ in place of $p_{j}$. Let $p_{↓}^{*}\left(i\right)$ and $p_{↓}^{*}\left(j\right)$ be the, respectively, re-arranged $p^{*}\left(i\right)$ and $p^{*}\left(j\right)$ in a non-increasing order. Then $K_{\alpha }\left(p_{↓}^{*}\left(i\right)\right)\leq K_{\alpha }\left(p_{↓}^{*}\left(j\right)\right)$.*

**Proof.** Without loss of generality, let it be assumed that the sequence $p=\{p_{k};k\geq 1\}$ is non-increasingly arranged. Denote $K_{\alpha }\left(p\right)=k_{\alpha }$ and note that

(A5) $$\sum_{i=k_{\alpha }}^{\infty }p_{\left(i\right)}\geq \alpha \;\mathrm{and}\;\sum_{i=k_{\alpha }+1}^{\infty }p_{\left(i\right)}<\alpha .$$

The lemma is established, respectively, in four exhaustive scenarios: (a) $i<j\leq k_{\alpha }$, (b) $i<k_{\alpha }\leq j$, (c) $i=k_{\alpha }<j$, and (d) $k_{\alpha }<i<j$. In scenario (a), the tail sequence $\left\{p_{k_{\alpha }},\cdots \right\}$ of p is preserved in $p_{↓}^{*}\left(i\right)$ and $p_{↓}^{*}\left(j\right)$ after adding a mass ε to $p_{i}$ or $p_{j}$, respectively. It follows that $K_{\alpha }\left(p_{↓}^{*}\left(i\right)\right)=K_{\alpha }\left(p_{↓}^{*}\left(j\right)\right)$ and hence $K_{\alpha }\left(p_{↓}^{*}\left(i\right)\right)\leq K_{\alpha }\left(p_{↓}^{*}\left(j\right)\right)$ holds. In scenario (b), the tail sequence $\left\{p_{k_{\alpha }},\cdots \right\}$ of p is preserved in $p_{↓}^{*}\left(i\right)$ after ε is added to $p_{i}$ and therefore $K_{\alpha }\left(p_{↓}^{*}\left(i\right)\right)=k_{\alpha }$. However, applying Corollary A1, it follows that $K_{\alpha }\left(p_{↓}^{*}\left(j\right)\right)\geq K_{\alpha }\left(p_{↓}\right)=k_{\alpha }$. Hence $K_{\alpha }\left(p_{↓}^{*}\left(i\right)\right)\leq K_{\alpha }\left(p_{↓}^{*}\left(j\right)\right)$ holds. In scenario (c), $p_{i}^{*}=p_{i}+\varepsilon$ occupies a position in $p_{↓}^{*}\left(i\right)$ with an index less or equal to $k_{\alpha }$. This fact implies that the value at the $k_{\alpha }$ th position in $p_{↓}^{*}\left(i\right)$ is a value greater or equal to $p_{k_{\alpha }}$. By the definition of $K_{\alpha }$ in (A1), $K_{\alpha }\left(p_{↓}^{*}\left(i\right)\right)=k_{\alpha }=K_{\alpha }\left(p_{↓}\right)$. However, applying Corollary A1, $K_{\alpha }\left(p_{↓}^{*}\left(j\right)\right)\geq K_{\alpha }\left(p_{↓}\right)=k_{\alpha }$. Hence $K_{\alpha }\left(p_{↓}^{*}\left(i\right)\right)\leq K_{\alpha }\left(p_{↓}^{*}\left(j\right)\right)$ holds. In scenario (d), let the position occupied by $p_{i}^{*}=p_{i}+\varepsilon$ in $p_{↓}^{*}\left(i\right)$ be denoted as *s* and that by $p_{j}^{*}=p_{j}+\varepsilon$ in $p_{↓}^{*}\left(j\right)$ as *t*. Let it be recognized that s≤t. The following three exhaustive sub-scenarios need to be, respectively, considered: (d1) $k_{\alpha }\leq s\leq t$, (d2) $s<k_{\alpha }\leq t$, and (d3) $s\leq t\leq k_{\alpha }$ In scenario (d1), it follows that $\sum_{k\geq k_{\alpha }}p_{\left(k\right)}^{*}\left(i\right)=\sum_{k\geq k_{\alpha }}p_{k}+\varepsilon >\alpha +\varepsilon >\alpha$, and similarly that $\sum_{k\geq k_{\alpha }}p_{\left(k\right)}^{*}\left(j\right)=\sum_{k\geq k_{\alpha }}p_{k}+\varepsilon >\alpha +\varepsilon >\alpha$. If ε is such that

(A6) $$\begin{array}{cc} \sum_{k\geq k_{\alpha }+1}p_{\left(k\right)}^{*}\left(i\right) & =\sum_{k\geq k_{\alpha }+1}p_{\left(k\right)}+\varepsilon \geq \alpha , \end{array}$$

then $K_{\alpha }\left(p_{↓}^{*}\left(i\right)\right)\geq k_{\alpha }+1$ and therefore, by Corollary A1, $K_{\alpha }\left(p_{↓}^{*}\left(i\right)\right)=k_{\alpha }+1$. On the other hand, since $p_{i}>p_{j}$, (A6) implies

$$\begin{array}{cc} \sum_{k\geq k_{\alpha }+1}p_{\left(k\right)}^{*}\left(j\right) & =\sum_{k\geq k_{\alpha }+1}p_{\left(k\right)}+\varepsilon =\sum_{k\geq k_{\alpha }+1}p_{\left(k\right)}^{*}\left(i\right)\geq \alpha , \end{array}$$

which in turn implies that $K_{\alpha }\left(p_{↓}^{*}\left(j\right)\right)\geq k_{\alpha }+1$. By Corollary A1, $K_{\alpha }\left(p_{↓}^{*}\left(j\right)\right)=k_{\alpha }+1$ and therefore $K_{\alpha }\left(p_{↓}^{*}\left(i\right)\right)=K_{\alpha }\left(p_{↓}^{*}\left(j\right)\right)$. Hence $K_{\alpha }\left(p_{↓}^{*}\left(i\right)\right)\leq K_{\alpha }\left(p_{↓}^{*}\left(j\right)\right)$ holds. If

(A7) $$\begin{array}{cc} \sum_{k\geq k_{\alpha }+1}p_{\left(k\right)}^{*}\left(i\right) & =\sum_{k\geq k_{\alpha }+1}p_{\left(k\right)}+\varepsilon <\alpha , \end{array}$$

then $K_{\alpha }\left(p_{↓}^{*}\left(i\right)\right)\leq k_{\alpha }$ and therefore, by Corollary A1, $K_{\alpha }\left(p_{↓}^{*}\left(i\right)\right)=k_{\alpha }$. On the other hand, since $p_{i}>p_{j}$, (A7) implies

$$\begin{array}{cc} \sum_{k\geq k_{\alpha }+1}p_{\left(k\right)}^{*}\left(j\right) & =\sum_{k\geq k_{\alpha }+1}p_{\left(k\right)}+\varepsilon =\sum_{k\geq k_{\alpha }+1}p_{\left(k\right)}^{*}\left(i\right)<\alpha , \end{array}$$

which in turn implies that $K_{\alpha }\left(p_{↓}^{*}\left(j\right)\right)\leq k_{\alpha }$. By Corollary A1, $K_{\alpha }\left(p_{↓}^{*}\left(j\right)\right)=k_{\alpha }$ and therefore $K_{\alpha }\left(p_{↓}^{*}\left(i\right)\right)=K_{\alpha }\left(p_{↓}^{*}\left(j\right)\right)$. Hence $K_{\alpha }\left(p_{↓}^{*}\left(i\right)\right)\leq K_{\alpha }\left(p_{↓}^{*}\left(j\right)\right)$ holds. In scenario (d2), let it be noted first that

1. the value at the $k_{\alpha }+1$ st position in $p_{↓}^{*}\left(i\right)$ is $p_{k_{\alpha }}$ and the value at the $k_{\alpha }$ th position in $p_{↓}^{*}\left(j\right)$ is also $p_{k_{\alpha }}$; and
2. $p_{i}+\varepsilon >p_{k_{\alpha }}$ and therefore $\varepsilon >p_{k_{\alpha }}-p_{i}$.

Consider the two tail sums of $p_{↓}^{*}\left(i\right)$ and $p_{↓}^{*}\left(j\right)$. First for *i*,

(A8) $$\begin{array}{cc} \tau_{k_{\alpha }+1}^{*}\left(i\right) & =\sum_{k\geq k_{\alpha }+1}p_{\left(k\right)}^{*}\left(i\right)=\sum_{k\geq k_{\alpha }}p_{k}-p_{i},\\ \tau_{k_{\alpha }+2}^{*}\left(i\right) & =\sum_{k\geq k_{\alpha }+2}p_{\left(k\right)}^{*}\left(i\right)=\sum_{k\geq k_{\alpha }+1}p_{k}-p_{i}<\alpha -p_{i}<\alpha ; \end{array}$$

and next for *j*,

(A9) $$\begin{array}{cc} \tau_{k_{\alpha }}^{*}\left(j\right) & =\sum_{k\geq k_{\alpha }}p_{\left(k\right)}^{*}\left(j\right)=\sum_{k\geq k_{\alpha }}p_{k}+\varepsilon >\alpha +\varepsilon >\alpha ,\\ \tau_{k_{\alpha }+1}^{*}\left(j\right) & =\sum_{k\geq k_{\alpha }+1}p_{\left(k\right)}^{*}\left(j\right)=\sum_{k\geq k_{\alpha }+1}p_{k}+\varepsilon >\sum_{k\geq k_{\alpha }+1}p_{k}+p_{k_{\alpha }}-p_{i}=\sum_{k\geq k_{\alpha }}p_{k}-p_{i}\geq \alpha . \end{array}$$

If $\tau_{k_{\alpha }+1}^{*}\left(i\right)\geq \alpha$, then by (A8) $K_{\alpha }\left(p_{↓}^{*}\left(i\right)\right)=k_{\alpha }+1$. On the other hand, by (A9), $K_{\alpha }\left(p_{↓}^{*}\left(j\right)\right)\geq k_{\alpha }+1$. By Corollary A1, $K_{\alpha }\left(p_{↓}^{*}\left(j\right)\right)=k_{\alpha }+1$, and hence $K_{\alpha }\left(p_{↓}^{*}\left(j\right)\right)\geq K_{\alpha }\left(p_{↓}^{*}\left(i\right)\right)$ holds. If $\tau_{k_{\alpha }+1}^{*}\left(i\right)<\alpha$, then, by Corollary A1, $K_{\alpha }\left(p_{↓}^{*}\left(i\right)\right)=k_{\alpha }$. Additionally, by Corollary A1, $K_{\alpha }\left(p_{↓}^{*}\left(j\right)\right)\geq k_{\alpha }$, and hence $K_{\alpha }\left(p_{↓}^{*}\left(j\right)\right)\geq K_{\alpha }\left(p_{↓}^{*}\left(i\right)\right)$ holds. In scenario (d3), let it be noted first that both values at the $k_{\alpha }+1$ st position in $p_{↓}^{*}\left(i\right)$ and at the $k_{\alpha }+1$ st position in $p_{↓}^{*}\left(j\right)$ ar, respectively, $p_{k_{\alpha }}$. Consider the two tail sums of $p_{↓}^{*}\left(i\right)$ and $p_{↓}^{*}\left(j\right)$. First for *i*,

(A10) $$\begin{array}{cc} \tau_{k_{\alpha }+1}^{*}\left(i\right) & =\sum_{k\geq k_{\alpha }+1}p_{\left(k\right)}^{*}\left(i\right)=\sum_{k\geq k_{\alpha }}p_{k}-p_{i},\\ \tau_{k_{\alpha }+2}^{*}\left(i\right) & =\sum_{k\geq k_{\alpha }+2}p_{\left(k\right)}^{*}\left(i\right)=\sum_{k\geq k_{\alpha }+1}p_{k}-p_{i}<\alpha -p_{i}<\alpha ; \end{array}$$

and next for *j*,

(A11) $$\begin{array}{cc} \tau_{k_{\alpha }+1}^{*}\left(j\right) & =\sum_{k\geq k_{\alpha }+1}p_{\left(k\right)}^{*}\left(j\right)=\sum_{k\geq k_{\alpha }}p_{k}-p_{j},\\ \tau_{k_{\alpha }+2}^{*}\left(j\right) & =\sum_{k\geq k_{\alpha }+2}p_{\left(k\right)}^{*}\left(j\right)=\sum_{k\geq k_{\alpha }+1}p_{k}-p_{j}<\alpha -p_{j}<\alpha . \end{array}$$

If $\tau_{k_{\alpha }+1}^{*}\left(i\right)\geq \alpha$, then by (A10) $K_{\alpha }\left(p_{↓}^{*}\left(i\right)\right)=k_{\alpha }+1$. On the other hand, since $p_{i}>p_{j}$, $\tau_{k_{\alpha }+1}^{*}\left(i\right)\geq \alpha$ implies $\tau_{k_{\alpha }+1}^{*}\left(j\right)\geq \alpha$, it follows by (A11) that $K_{\alpha }\left(p_{↓}^{*}\left(j\right)\right)=k_{\alpha }+1$. Therefore $K_{\alpha }\left(p_{↓}^{*}\left(j\right)\right)\geq K_{\alpha }\left(p_{↓}^{*}\left(i\right)\right)$ holds. If $\tau_{k_{\alpha }+1}^{*}\left(i\right)<\alpha$, then by Corollary A1, $K_{\alpha }\left(p_{↓}^{*}\left(i\right)\right)=k_{\alpha }$. However $\tau_{k_{\alpha }+1}^{*}\left(j\right)$ may take a value less than α or greater or equal to α. In the first case, it is implied that $K_{\alpha }\left(p_{↓}^{*}\left(j\right)\right)=k_{\alpha }$, that is, $K_{\alpha }\left(p_{↓}^{*}\left(j\right)\right)\geq K_{\alpha }\left(p_{↓}^{*}\left(i\right)\right)$ holds. Or in the second case, it is implied that $K_{\alpha }\left(p_{↓}^{*}\left(j\right)\right)=k_{\alpha }+1$, which in turn implies $K_{\alpha }\left(p_{↓}^{*}\left(j\right)\right)\geq K_{\alpha }\left(p_{↓}^{*}\left(i\right)\right)$ holds. At this point, the claim of the lemma is established for all scenarios and sub-scenarios. □

**Proposition** **A1.**
*Let $p=\{p_{k};k\geq 1\}$ be a probability distribution on a countable alphabet and let $p_{↓}=\left\{p_{\left(k\right)};k\geq 1\right\}$ be a non-increasing arranged p. Suppose, for two particular indices i and j such that i<j, a mass of ε>0 is transferred from $p_{\left(i\right)}$ to $p_{\left(j\right)}$, subject to $0<\varepsilon <p_{\left(i\right)}-p_{\left(j\right)}$. Let the sequence after the transfer be denoted as $p^{*}$ and its non-increasingly re-arranged version as $p_{↓}^{*}$. Then for any α∈(0,1), $K_{\alpha }\left(p_{↓}\right)\leq K_{\alpha }\left(p_{↓}^{*}\right)$.*

**Proof.** Without loss of generality, let it be assumed that $p=\{p_{k};k\geq 1\}$ is non-increasingly arranged. Since $K_{\alpha }\left(p_{↓}\right)$ is symmetric with respect to *i* and *j*, it suffices to show that $K_{\alpha }\left(p_{↓}\right)\leq K_{\alpha }\left(p_{↓}^{*}\right)$ for any transfer of ε mass for $\varepsilon \in (0,\left(p_{i}-p_{j}\right)/2]$. Toward that end, consider the following sequence of non-negative values,

$$\begin{array}{cc} p^{-\varepsilon } & =\{p_{1},\cdots ,p_{i-1},p_{i}-\varepsilon ,p_{i+1},\cdots ,p_{j-1},p_{j},p_{j+1},\cdots \}. \end{array}$$

It is to be noted first that adding ε to $p_{i}-\varepsilon$ in $p^{-\varepsilon }$ gives p and second that adding ε to $p_{j}$ in $p^{-\varepsilon }$ gives

$$\begin{array}{cc} p^{*} & =\{p_{1},\cdots ,p_{i-1},p_{i}-\varepsilon ,p_{i+1},\cdots ,p_{j-1},p_{j}+\varepsilon ,p_{j+1},\cdots \}. \end{array}$$

Since $p_{i}-\varepsilon \geq p_{j}$, by Lemma A2, $K_{\alpha }\left(p_{↓}\right)\leq K_{\alpha }\left(p_{↓}^{*}\right)$. □

Before stating and proving Proposition A2 below, a simple and trivial fact is summarized in the following lemma for easy reference.

**Lemma** **A3** (Stairway Carpeting)**.** *Let $q=\{q_{k};k\geq 1\}$ be a sequence of non-increasingly ordered non-negative values such that (a) $q_{1}>0$ and (b) $\sum_{k\geq 1}q_{k}=C>0$. Let ε>0 be any positive value. Then there exists a sequence of non-negative values $\varepsilon =\{\varepsilon_{k};k\geq 1\}$ satisfying $\sum_{k\geq 1}\varepsilon_{k}=\varepsilon$, such that, letting $q^{*}=\left\{q_{k}^{*};k\geq 1\right\}$ where $q_{k}^{*}=q_{k}+\varepsilon_{k}$,*

1. *$q_{k}^{*}\geq q_{k+1}^{*}$ for each and every k≥1, and*
2. *$\sum_{k\geq 1}q_{k}^{*}=C+\varepsilon$.*

**Proof.** Since (a) and (b), there exists an index value $k_{0}$ such that $q_{k_{0}}>q_{k_{0}+1}$ and hence $q_{k_{0}}-q_{k_{0}+1}>0$. Let M=M(q,ε) be an integer such that $\varepsilon /M\leq q_{k_{0}}-q_{k_{0}+1}$. Let $\varepsilon_{k}=0$ for $k=1,\cdots ,k_{0}$, $\varepsilon_{k}=\varepsilon /M$ for $k=k_{0}+1,\cdots ,k_{0}+M$, and $\varepsilon_{k}=0$ for $k\geq k_{0}+M+1$. It can be easily verified that the claim of the lemma holds. □

Lemma A3 has the following two important implications that are relevant in the proof of Proposition A2.

1. For any ordered non-negative sequence, there exists a way to distribute any additional non-negative mass on top of the sequence and yet to preserve the non-increasing order of the sequence. Any such existent way will be referred to as a way of Stairway Carpeting.
2. A transfer of mass ε to q by a way of Stairway Carpeting may be viewed as a sequence of *M* steps, in each of which a part of ε, ε/M, is transferred.

**Proposition** **A2.**
*The generalized species richness, $K_{\alpha }$, is 100×α%-robust.*

**Proof.** Consider any given probability distribution $p=\{p_{k};k\geq 1\}$, which can be without loss of generality assumed to be non-increasingly arranged, a given α∈(0,1) and a given ε∈(0,α). An ε-mass re-distribution of p is a combination of two steps: (a) an arbitrarily reduction of ε mass from p and (b) an arbitrarily add-back of the same ε mass. Let the reduction, the add-back and their differences be represented by

$$\varepsilon_{1}=\left\{\varepsilon_{1,k};k\geq 1\right\},\;\varepsilon_{2}=\left\{\varepsilon_{2,k};k\geq 1\right\}\mathrm{and}\;\delta =\left\{\delta_{k};k\geq 1\right\}$$

where $\varepsilon_{i,k}\geq 0$ and $\sum_{k\geq 1}\varepsilon_{i,k}=\varepsilon$ for i=1,2, $\delta_{k}=\varepsilon_{2,k}-\varepsilon_{1,k}$ for each *k* and $\sum_{k\geq 1}\delta_{k}=0$. Let the distribution after the re-distribution be denoted as

(A12) $$p^{*}=\left\{p_{k}^{*};k\geq 1\right\}=\left\{p_{k}+\delta_{k};k\geq 1\right\}.$$

For any ε∈(0,α), it is desired to show that $K_{\alpha }\left(p^{*}\right)$ is bounded above by a constant only depending on p, α and ε (but not on $\varepsilon_{1}$ and $\varepsilon_{2}$). Toward that end, let it first be noted that $k_{\alpha -\varepsilon }=K_{\alpha -\varepsilon }\left(p\right)$ is a constant integer only depending on p, α and ε. Several modifications are to be made to $p^{*}$. First let all $\delta_{k}<0$ be set to zero, that is, let $\delta_{k}^{*}=\max\left\{\delta_{k},0\right\}$ and write the modified $p^{*}$ as

(A13) $$p_{1}^{*}=\left\{p_{k}+\delta_{k}^{*};k\geq 1\right\}=\left\{\left(p_{1}+\delta_{1}^{*}\right),\cdots ,\left(p_{k_{\alpha -\varepsilon }}+\delta_{k_{\alpha -\varepsilon }}^{*}\right),\left(p_{k_{\alpha -\varepsilon }+1}+\delta_{k_{\alpha -\varepsilon }+1}^{*}\right),\cdots \right\}.$$

By Corollary A1, it follows that

(A14) $$K_{\alpha }\left(p^{*}\right)\leq K_{\alpha }\left(p_{1}^{*}\right).$$

Let is be observed that there are only finitely many terms in $p_{1}^{*}$ of (A13) that are greater than or equal to $p_{k_{\alpha -\varepsilon }}$. Each of these terms corresponds to an index *k*. Let the maximum of these indices be denoted as $k_{0}$ (so that, for each $k\geq k_{0}+1$, $p_{k}+\delta_{k}^{*}<p_{k_{\alpha -\varepsilon }}$ and that $k_{0}\geq k_{\alpha -\varepsilon }$). Second, let $p_{1}^{*}$ be further modified in such a way that the first $k_{0}$ terms are preserved but the remainder terms, from $k=k_{0}+1$ on, are re-arranged into a non-increasing order. Denote the resulting sequence by

(A15) $$p_{2}^{*}=\left\{\left(p_{1}+\delta_{1}^{*}\right),\cdots ,\left(p_{k_{0}}+\delta_{k_{0}}^{*}\right),p_{k_{0}+1}^{**},\cdots \right\}.$$

Since $K_{\alpha }$ is permutation invariant, it follows that

(A16) $$K_{\alpha }\left(p^{*}\right)\leq K_{\alpha }\left(p_{1}^{*}\right)=K_{\alpha }\left(p_{2}^{*}\right).$$

Next, for each $k=1,\cdots ,k_{0}$, collect $\delta_{k}^{*}$ from $p_{k}+\delta_{k}^{*}$ and re-distribute the mass of $\varepsilon =\delta_{k}^{*}$ to the tail sequence of $p_{2}^{*}$, $q=\{p_{k_{0}+1}^{**},p_{k_{0}+2}^{**},\cdots \}$, by means of a Stairway Carpeting way described in Lemma A3. The resulting sequence would have the following form

(A17) $$p_{3}^{*}=\left\{p_{k}^{***};k\geq 1\right\}=\left\{p_{1},\cdots ,p_{k_{\alpha -\varepsilon }},p_{k_{\alpha -\varepsilon }+1},\cdots ,p_{k_{0}},p_{k_{0}+1}^{***},\cdots \right\}.$$

By construction, $p_{3}^{*}$ satisfies the following three properties:

1. the sub-sequence $\left\{p_{1},\cdots ,p_{k_{0}}\right\}$ is non-increasingly ordered;
2. each term in the tail sequence $\left\{p_{k_{0}+1}^{***},\cdots \right\}$ is less than $p_{k_{\alpha -\varepsilon }}$; and
3. the sum of all terms in $p_{3}^{*}$ from $k=k_{\alpha -\varepsilon }+1$ on is
  (A18) $$\sum_{k\geq k_{\alpha -\epsilon }+1}p_{k}^{***}=\sum_{k\geq k_{\alpha -\epsilon }+1}p_{k}+\sum_{k\geq 1}\delta_{k}^{*}<\left(\alpha -\varepsilon \right)+\sum_{k\geq 1}\delta_{k}^{*}<\alpha .$$

By (A18) and the definition of $K_{\alpha }$,

(A19) $$K_{\alpha }\left(p_{3}^{*}\right)\leq K_{\alpha -\varepsilon }\left(p\right)=k_{\alpha -\varepsilon }.$$

Finally, let it be noted that the modification of (A15) into (A17) is a finite sequence of steps each of which transfers a probability from a higher term to a lower term—the second implication of Lemma A2 mentioned above. Applying Corollary A1 as finitely many times as needed, it follows that

(A20) $$K_{\alpha }\left(p_{2}^{*}\right)\leq K_{\alpha }\left(p_{3}^{*}\right).$$

Combining (A16), (A19) and (A20) gives $K_{\alpha }\left(p^{*}\right)\leq k_{\alpha -\varepsilon }<\infty$. □
